# Enolates ambushed – asymmetric tandem conjugate addition and subsequent enolate trapping with conventional and less traditional electrophiles

**DOI:** 10.3762/bjoc.19.44

**Published:** 2023-05-04

**Authors:** Péter Kisszékelyi, Radovan Šebesta

**Affiliations:** 1 Department of Organic Chemistry, Faculty of Natural Sciences, Comenius University Bratislava, Mlynská dolina, Ilkovičova 6, 842 15 Bratislava, Slovakia

**Keywords:** asymmetric catalysis, conjugate addition, electrophile, enolate, tandem reaction

## Abstract

Metal enolates are useful intermediates and building blocks indispensable in many organic synthetic transformations. Chiral metal enolates obtained by asymmetric conjugate additions of organometallic reagents are structurally complex intermediates that can be employed in many transformations. In this review, we describe this burgeoning field that is reaching maturity after more than 25 years of development. The effort of our group to broaden possibilities to engage metal enolates in reactions with new electrophiles is described. The material is divided according to the organometallic reagent employed in the conjugate addition step, and thus to the particular metal enolate formed. Short information on applications in total synthesis is also given.

## Introduction

**Scheme 1:**
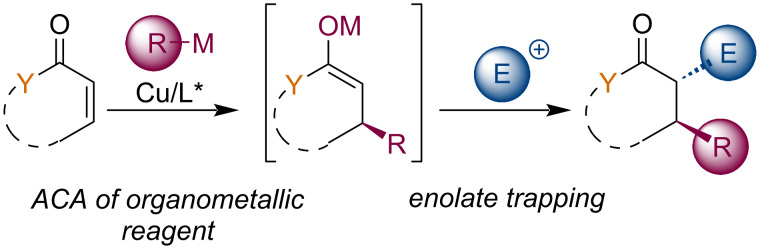
General scheme depicting tandem reactions based on an asymmetric conjugate addition followed by an enolate trapping.

The formation of complex chiral molecules is a crucial task of organic synthesis that enables the synthesis of pharmaceuticals, crop-protecting agents, or advanced materials. Their syntheses often involve numerous reaction steps requiring laborious isolation and intermediate product purification steps. An important strategy for improving syntheses’ effectiveness is the concept of domino reactions, cascade, or tandem reactions. These transformations combine several reactions into a sequence that uses functionalities generated in previous steps without isolating intermediates [1]. Stabilized carbon-based nucleophiles, or in other words, conjugate bases of weak C–H acids, are termed enolates, and they participate in a large array of organic synthetic transformations. Enolates are usually formed by deprotonation of the corresponding organic compound. However, other synthetic approaches for their generation exist, such as cleavage of enol ethers and esters, halogen–metal exchange, transmetalations, and conjugate additions to α,β-unsaturated carbonyl compounds [2]. In particular, the last-mentioned method is highly synthetically relevant. This approach has the advantage of being more selective and affording more molecular complexity in one step. In addition, transition-metal catalysis allows the introduction of stereogenic information, thus leading to chiral products. Enolate species are uniquely positioned for reactivity with a broad array of electrophiles and thus allowing quick and efficient construction of highly complex structures from readily available starting materials. Various polar organometallic reagents were successfully employed in asymmetric conjugate additions (ACA) [3–9], mainly organozinc [10], Grignard [11–13], trialkylaluminum [14], or organozirconium reagents [15]. Additions with these reagents lead to corresponding zinc, magnesium, aluminum, and zirconium enolates, which all possess helpful and, to an extent, specific reactivity characteristics. Interesting boron and silicon enolates can be generated by asymmetric conjugate boration [16], or silylation [17]. From several potentially catalytically active transition metals, copper combines beneficial properties for both activation of the Michael acceptor and the formation of intermediate organocuprates from stoichiometric organometallic reagents [18]. Metal enolates formed in this way can react in many transformations (Scheme 1) [19–20]. It has been documented that metal enolates from conjugate additions engaged in aldol, and Mannich-type reactions, Michael addition, nucleophilic substitutions, cyclopropanations, and reactions with carbocations. The field of asymmetric conjugate addition with its extension into enolate trapping reactions began to develop approximately in 1996. In this review article, we analyze more recent realizations of this strategy focusing on lesser-studied trapping reactions and works after 2010. We also present here our attempts to broaden the scope of these enolate trapping reactions by using different types of electrophilic reagents.

## Review

### Conjugate additions with organozinc reagents

**Scheme 2:**
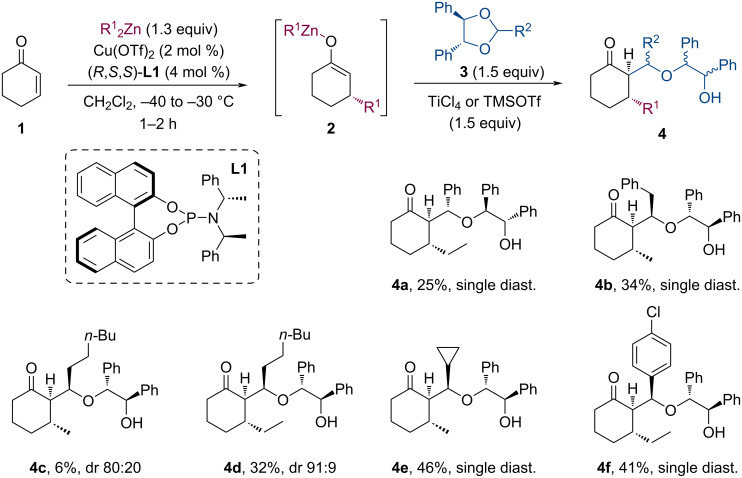
Cu-catalyzed tandem conjugate addition of R_2_Zn/aldol reaction with chiral acetals.

Following the seminal work of Feringa in 1997 [21], the tandem asymmetric organozinc conjugate addition followed by subsequent aldol reaction was scarcely applied in the last decade. Welker and Woodward studied the reaction of zinc enolates **2** with chiral acetals **3** (Scheme 2) [22]. The Lewis acid (TiCl_4_ or TMSOTf) promoted trapping gave the aldol adducts **4** in good to excellent diastereoselectivity (up to a single diastereomer), but the yields were relatively low (25–44%). To overcome this limitation, the authors used TMSOTf to prepare and isolate the corresponding silyl enol ethers, which were later successfully applied in the Mukiyama aldol reaction to gain the originally desired aldol adducts with improved yields and still good dr. Finally, the cerium ammonium nitrate (CAN) promoted one-step oxidative removal of the chiral auxiliary group was also successfully demonstrated.

**Scheme 3:**
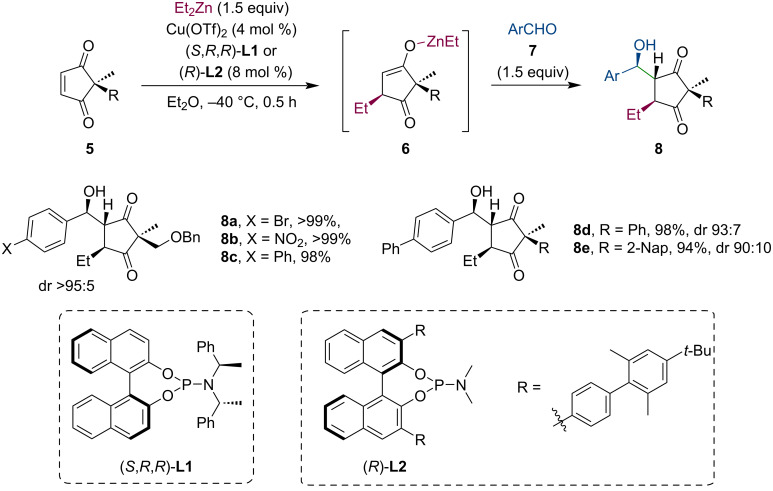
Cu-catalyzed asymmetric desymmetrization of cyclopentene-1,3-diones using a tandem conjugate addition/aldol reaction sequence.

In 2012, Aikawa et al. presented their work on the asymmetric desymmetrization of cyclopentene-1,3-diones **5** (Scheme 3) [23]. Following the Cu(OTf)_2_-catalyzed conjugate addition of R_2_Zn, the enolate **6** was trapped by several aromatic aldehydes **7**. These complex chiral cyclopentane derivatives **8** bearing all-carbon quaternary stereocenters were isolated in excellent yields and high diastereoselectivity. The authors have shown that catalyst loadings as low as 0.5 mol % can still be sufficient to promote the highly stereoselective reaction.

**Scheme 4:**
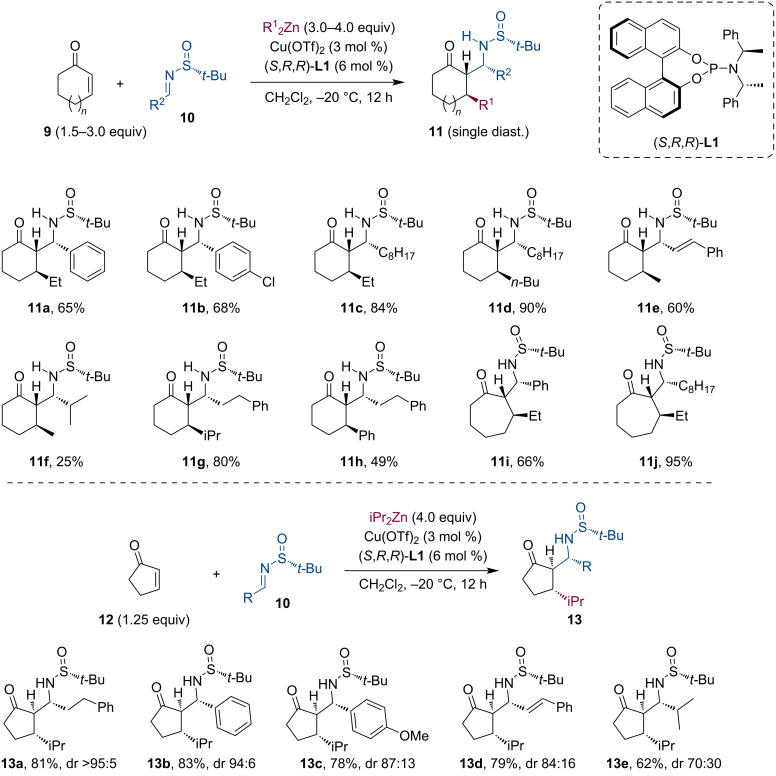
Stereocontrolled assembly of dialkylzincs, cyclic enones, and sulfinylimines utilizing a Cu-catalyzed tandem conjugate addition/Mannich reaction sequence.

Similarly to aldol reactions, Mannich-type additions are also suitable to trap the metal enolate. González-Gómez et al. studied the tandem conjugate addition of dialkylzincs to cyclic enones (**9**, **12**) and the subsequent reaction of the enolate with *N*-*tert*-butanesulfinylimines **10** (Scheme 4) [24–26]. Their method was applied to a broad range of substrates (5–7-membered rings) with equally high diastereoselectivity and good to excellent yields. In most cases, the authors detected only a single diastereomer in the crude reaction mixture (NMR). Using the enantiomeric form of the ligand or the chiral sulfoximine reagent, four diastereomeric β-aminoketones can be produced in excellent enantiomeric purity. Further transformations of the products were demonstrated in several examples, including reduction, acidic deprotection and subsequent base-mediated cyclization, or Baeyer–Villiger oxidation.

**Scheme 5:**
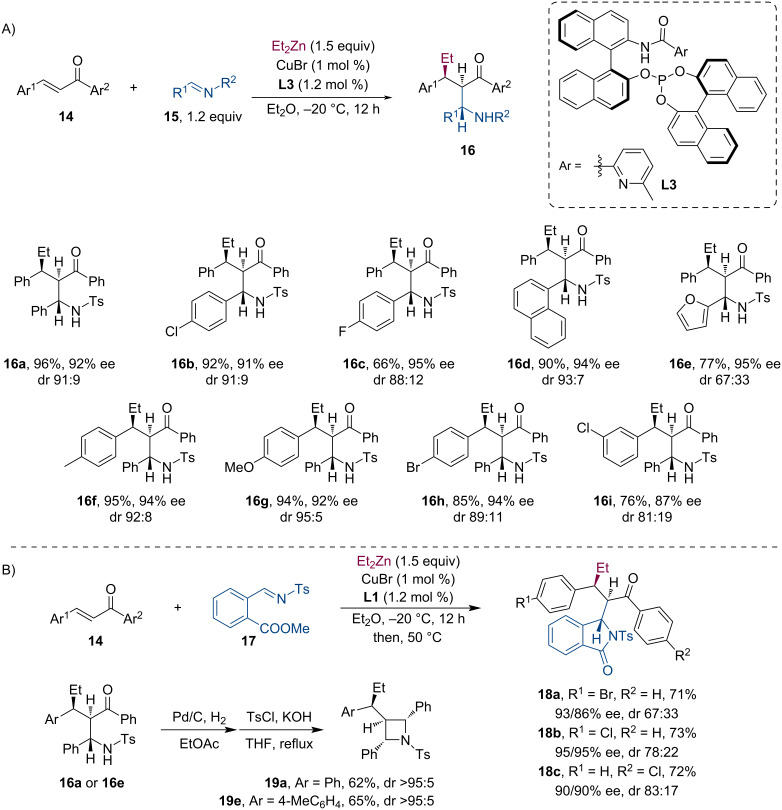
Cu-catalyzed tandem conjugate addition/Mannich reaction (A). Access to chiral isoindolinones and trisubstituted azetidines with contiguous stereocenters (B).

At about the same time, Huang and co-workers have developed similar asymmetric tandem sequences using acyclic enones **14** [27]. Their tandem conjugate addition/Mannich reaction methodology offers access to various non-cyclic β-aminoketones **16** with multiple contiguous stereocenters in high diastereo- and enantioselectivity (Scheme 5a). Additionally, chiral isoindolinones **18** and 2,3,4-trisubstituted azetidines **19** were also synthesized using this methodology (Scheme 5b).

**Scheme 6:**
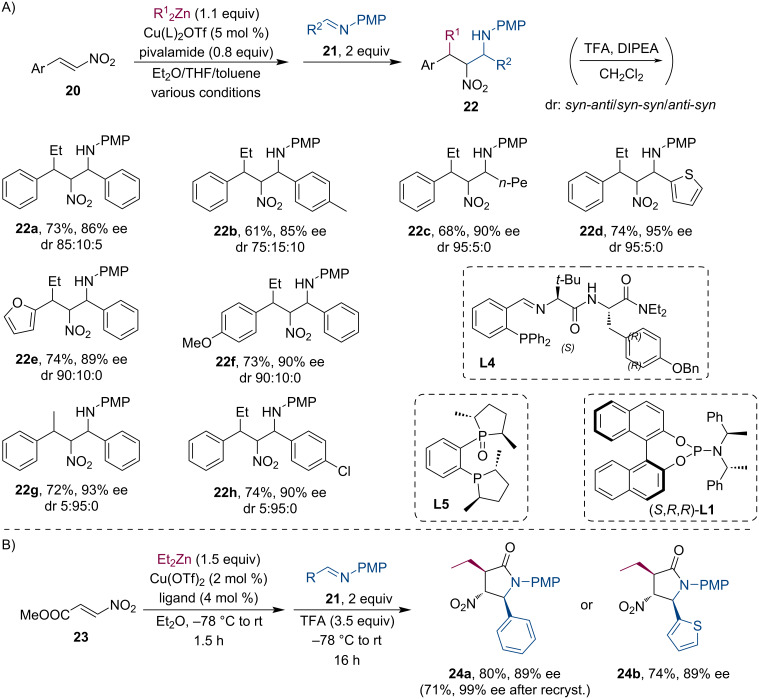
Cu-catalyzed tandem conjugate addition/nitro-Mannich reaction (A) with *syn*–*anti* or *syn*–*syn* selectivity (the products were isolated as trifluoroacetamide). Additional in situ lactamization results in nitropyrrolidinones with excellent stereoselectivity (B).

Nitronate anions were also found suitable for Mannich-type trapping reactions [28–29]. Anderson and co-workers accomplished several Cu-catalyzed conjugate additions of R_2_Zn to nitroolefins **20**, followed by subsequent reaction with *p*-methoxyphenyl (PMP)-protected imines **21** (Scheme 6A). By varying the reaction conditions, the *syn-anti* and the *syn*-*syn* diastereomers can be prepared with good yields and excellent stereoselectivity. Using nitroacrylate **23**, the authors have also demonstrated a tandem conjugate addition/nitro-Mannich/lactamization three-step reaction sequence resulting in trisubstituted nitropyrrolidinones **24** with exceptional enantioselectivity (Scheme 6B).

**Figure 1:**
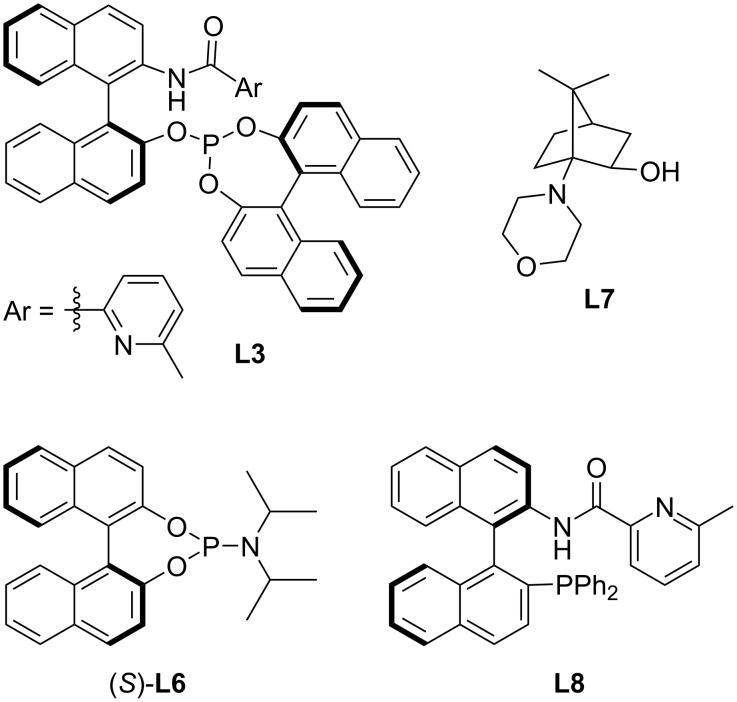
Various chiral ligands utilized for the tandem conjugate addition/Michael reaction sequences.

In contrast to conjugate additions to nitroolefins, these activated alkenes can also be utilized in the enolate trapping step. In the last decade, several highly stereoselective methodologies have been published that demonstrate the Cu- or Ni-catalyzed conjugate addition of organozincs to α,β-unsaturated ketones **14** followed by the reaction of the metal enolate with a nitroolefin (**20**) (Table 1) [30–33]. These reactions were facilitated by different ligand families (phosphite/phosphine-pyridine amide, phosphine-sulfoxide, phosphoramidite, MINBOL, see Figure 1) and they usually showed excellent diastereoselectivity (dr >20:1). The catalytic systems even with low catalyst loadings tolerated both electron-donating and withdrawing groups on the aromatic substituents. Therefore, numerous structurally distinct substrates were successfully utilized.

**Table 1:** Tandem reactions composed of ACA of R_2_Zn and enolate trapping with nitroalkenes.

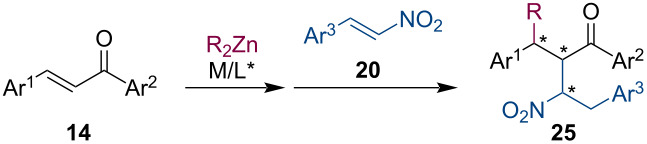					

Reference	Catalyst (mol %)	Ligand (mol %)	Conditions	Yield (%)	ee (%)

Huang, 2011 [30]	CuCl (1.0)	**L3** (1.2)	Et_2_O, − 20 °C, 24 h	52–90	91–97
Kang, 2011 [31]	Cu(OTf)_2_ (3.0)	**L6** (6.0)	toluene, −40 °C	25–89	76–96
Uang, 2015 [32]	Ni(acac)_2_ (0.5)	**L7** (12.5)	CH_3_CH_2_CN, −50 °C; then, 0 °C, 3 h	66–84	91–97
Hu, 2019 [33]	CuCl (2.0)	**L8** (2.5)	toluene, 0 °C, 12 h	60–88	90–97

**Scheme 7:**
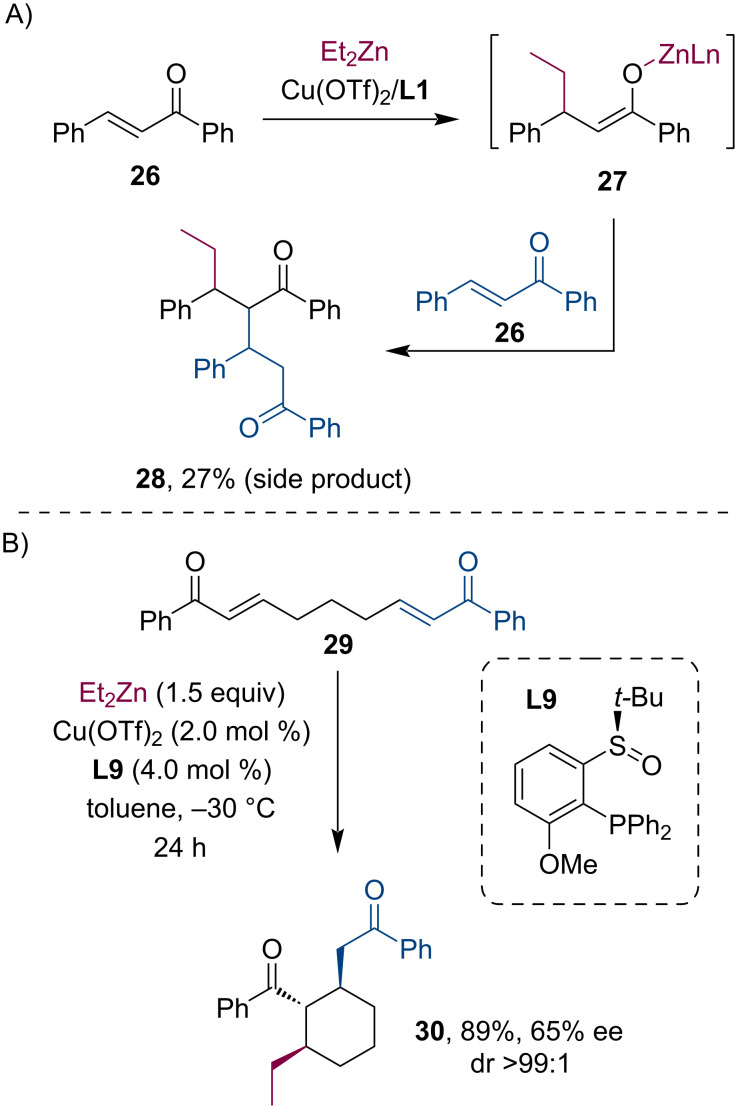
Cu-catalyzed tandem conjugate addition/Michael reaction: side-product formation with chalcone (A) and application to an intramolecular cyclization reaction (B).

Other than nitroolefins, Liao and co-workers have observed a side reaction of the α,β-unsaturated ketone **26** and the enolate **27** when they studied the conjugate addition of R_2_Zn reagents to chalcone and its derivatives (Scheme 7A) [34]. Encouraged by this, they have also attempted an intramolecular tandem conjugate addition/Michael reaction sequence, which has resulted in the expected cyclization product **30** in a diastereopure form (Scheme 7B).

**Scheme 8:**
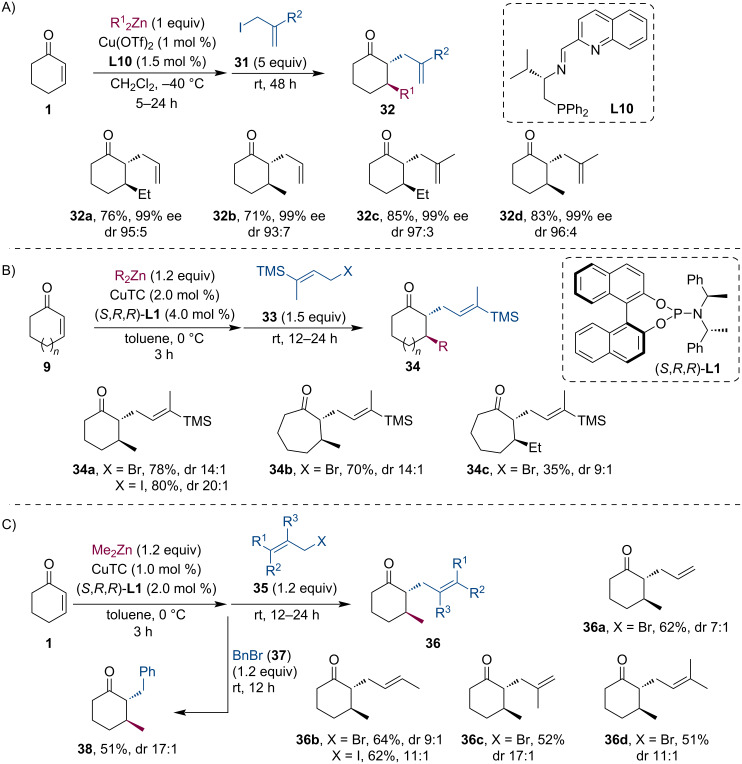
Zn enolate trapping using allyl iodides (A), Stork–Jung vinylsilane reagents (B), and allyl bromides or benzyl bromide (C).

Zinc enolates readily react with allyl iodides **31** or the structurally similar Stork–Jung vinylsilane reagents **33**. Kawamura et al. performed zinc enolate trapping reactions using ligand **L10**, a chiral quinoline-based N,N,P-ligand (Scheme 8A) [35]. The authors have concluded that the strict control of the amount of organozinc reagent added is essential to avoid side-product formation (diallylation) because the strongly basic R_2_Zn can form the enolate from the monoallylated product **32**. Therefore, using only 1 equiv of dialkylzinc, the desired allylated products **32** were isolated in good yields and excellent diastereo- and enantioselectivity. Soon after, Jarugumilli et al. investigated the enolate-trapping tandem sequence using various vinylsilanes **33** (Scheme 8B), allyl halides **35**, and benzyl bromide (**37**) (Scheme 8C) [36]. Although the asymmetric conjugate addition step routinely provided excellent selectivity (93–96% ee), only a moderate to good diastereomeric ratio was achieved.

**Scheme 9:**
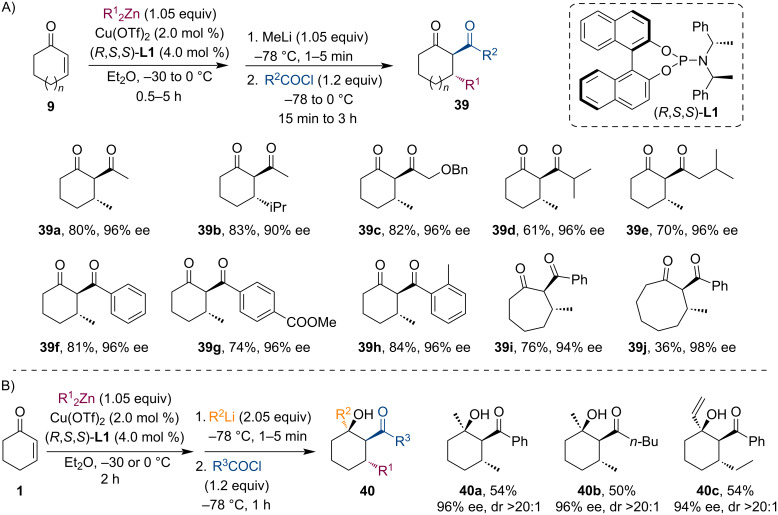
Cu-catalyzed tandem conjugate addition/acylation through Li R_2_Zn enolate (A). A four-component coupling reaction using nucleophilic trialkyl zincate (B).

Entrapping of the Zn enolate directly with acetyl chloride was found inefficient and led to a mixture of C-, O-, and diacylated products as described by Murphy and co-workers [37]. Encouraged by the work of Noyori on the activation of Li enolates using Me_2_Zn [38], they have tried to facilitate the enolate trapping by adding MeLi (1.05 equiv), which indeed led to a significant increase in yield and selectivity due to the high reactivity of the lithium dialkyl zincate enolate. Various 1,3-diketones **39** were prepared using this method with good yields and excellent enantioselectivities while only the *trans* diastereomers were detected (Scheme 9A). Furthermore, the authors have also demonstrated a four-component coupling reaction: by simply increasing the amount of the organolithium reagent (2.05 equiv) used for the activation of the Zn enolate, β-hydroxyketones **40** were gained via 1,2-addition of the zincate nucleophile (R_3_Zn^−^) to the ketone with moderate yields but still good stereoselectivities (Scheme 9B).

**Scheme 10:**
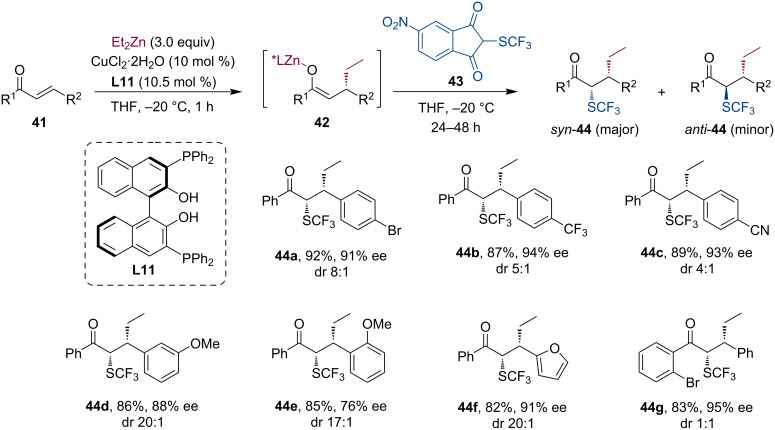
Selected examples for the Cu-catalyzed tandem conjugate addition/trifluoromethylthiolation sequence.

In 2018, Wang and co-workers extended the group of applicable electrophiles for the zinc enolate-based tandem reactions. Following the conjugate addition of Et_2_Zn to acyclic α,β-unsaturated ketones **41**, they have shown that several electrophilic SCF_3_ reagents (e.g., **43**) are suitable for enolate trapping (Scheme 10) [39]. This way, the strong electron-withdrawing SCF_3_ group can be efficiently introduced stereoselectively allowing access to structurally diverse compounds with altered pharmacochemical properties. In several cases the α-SCF_3_-substituted ketones **44** were isolated in good yields and enantioselectivities but with low diastereoselectivities.

Even though no asymmetric catalyst was involved, Kawano et al. recently demonstrated an attractive one-pot procedure for preparing complex bicyclic and bridged compounds utilizing catalytically generated bicyclic Zn enolates [40].

**Scheme 11:**
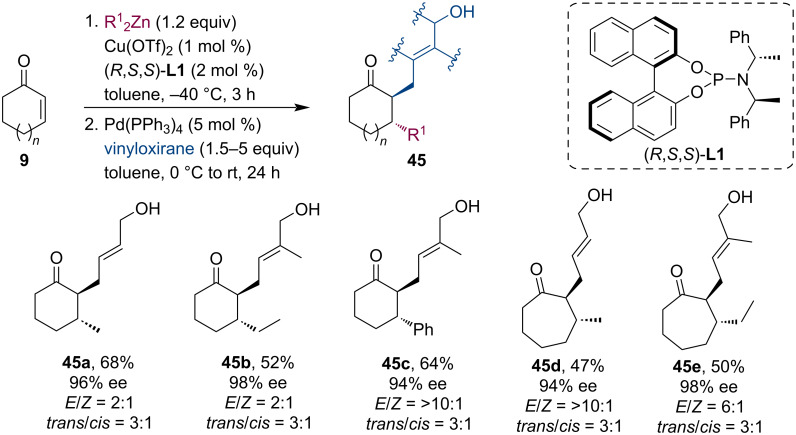
Zn enolates trapped by vinyloxiranes: synthesis of allylic alcohols.

Welker et al. have introduced the Pd-catalyzed trapping of zinc enolates with various vinyloxiranes [41]. This way, several allylic alcohols **45** were synthesized with moderate yields and excellent enantioselectivities (up to 98%) but low *trans*/*cis* selectivity (Scheme 11). Organoaluminum reagents (Me_3_Al, Et_3_Al) were also compatible with the reaction, however, they gave lower yields than the corresponding organozincs. The authors have also shown that these products are suitable for forming [6,7]-bicyclic adducts.

### Conjugate addition with Grignard reagents

**Scheme 12:**
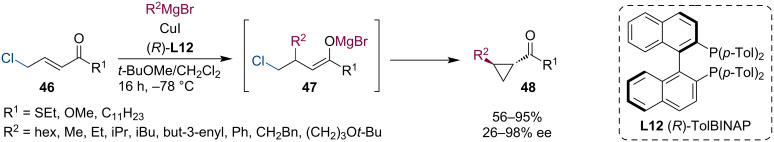
Stereoselective cyclopropanation of Mg enolates formed by ACA of Grignard reagents to chlorocrotonates.

Feringa and co-workers realized the tandem conjugate addition of Grignard reagents to 4-chlorocrotonates **46** [42]. The enolate **47**, which was formed in this process, underwent an intramolecular nucleophilic substitution to form cyclopropane derivatives. Thioesters, esters as well as ketones were compatible with this process. The chiral ligand **L12** afforded the highest enantioselectivities of up to 98% ee (Scheme 12).

**Scheme 13:**
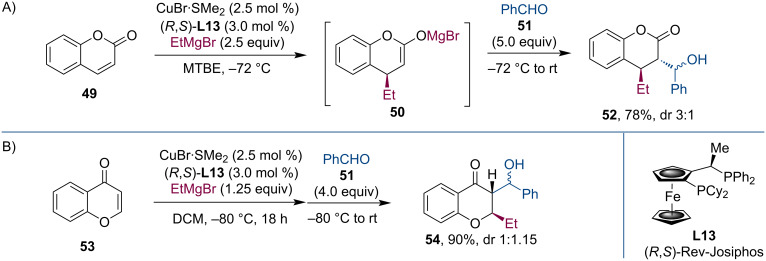
Domino aldol reactions of Mg enolates formed from coumarin and chromone.

Conjugate addition of Grignard reagents to coumarin (**49**) generated the corresponding magnesium enolates **50** [43]. In one instance, this enolate was trapped by benzaldehyde (**51**) (Scheme 13a). Related to this work, Feringa´s team realized also the conjugate addition to chromone (**53**) [44]. The enolate was again trapped with benzaldehyde in an aldol reaction (Scheme 13b).

**Scheme 14:**
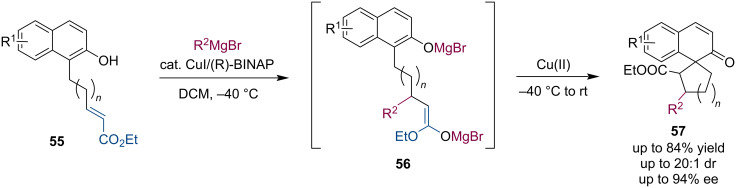
Oxidative coupling of ACA-produced Mg enolates.

Naphthol derivatives **55** bearing an α,β-unsaturated ester group undergo a copper(I)-catalyzed asymmetric conjugate addition. The magnesium enolates **56** then participated in a copper(II)-mediated intramolecular oxidative coupling to afford benzofused spirocyclic cycloalkanones **57** (Scheme 14) [45].

Our team became interested in domino reactions of metal enolates generated by Cu-catalyzed asymmetric conjugate additions of Grignard reagents. At the outset of our studies, there were works in which dialkylzinc additions were utilized to generate zinc enolates, and these enolates were then trapped with chiral sulfonylimines [24]. Specifically, we asked whether these magnesium enolates could be trapped with imines or their synthetic equivalents. Furthermore, we wanted to develop an enantioselective and diastereoselective process without adding chirality elements within the reagents.

**Scheme 15:**
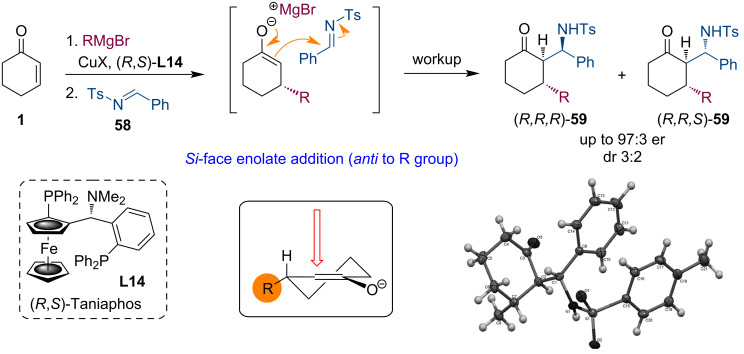
Tandem ACA of Grignard reagents to enones and Mannich reaction.

For our initial studies, we have selected the well-studied cyclic enones as substrates and the Taniaphos ligand (**L14**) that has been shown to impart high levels of enantioselectivity for these ketones [46]. We performed the conjugate addition for 2 h and then added imine **58** having a tosyl protecting group. The workup allowed the isolation of domino products **59** as a mixture of diastereomers with dr 3:2 and enantiomeric purities up to 97:3 er (Scheme 15) [47]. These experiments showed that the concept of interception of magnesium enolates, derived from Cu-ACA, with imines can be realized. As it could have been predicted, chiral enolates reacted with high diastereoselectivity with their *Si*-face (attack *anti* to the R group introduced during the conjugate addition). On the other hand, a typical problem of these reactions was also revealed. The diastereoselectivity with respect to the addition to the imine was only very modest.

**Scheme 16:**
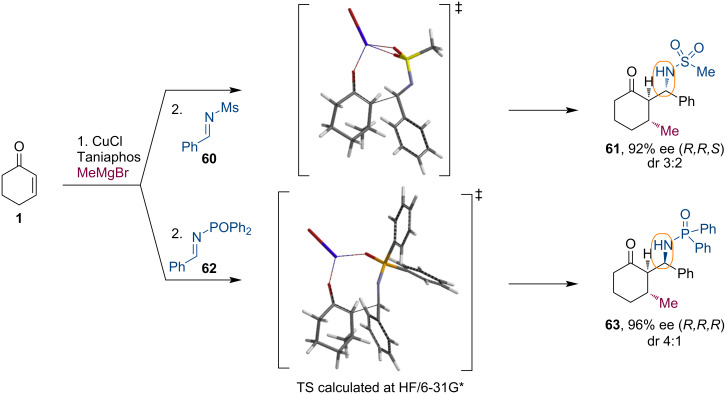
Diastereodivergent Mannich reaction of Mg enolates with differently *N*-protected imines.

To address the problem of low facial selectivity of the imine addition, we continued our study with several imines bearing various *N*-protecting groups [48]. We have argued that this protecting group could influence the enolate addition. Indeed, an effect of the nitrogen protecting group was observed. Interestingly, small sulfonyl-based protecting groups led to the (*R,R,S*)-diastereoisomer of the product **61**. On the other hand, the sterically bulky diphenylphosphorane group afforded the (*R,R,R*)-diastereoisomer **63** as the main product. The large protecting group likely overrides the repulsive interaction between the enolate and a phenyl group in a preferred synclinal Mg-bound arrangement of the reagents (Scheme 16).

**Scheme 17:**
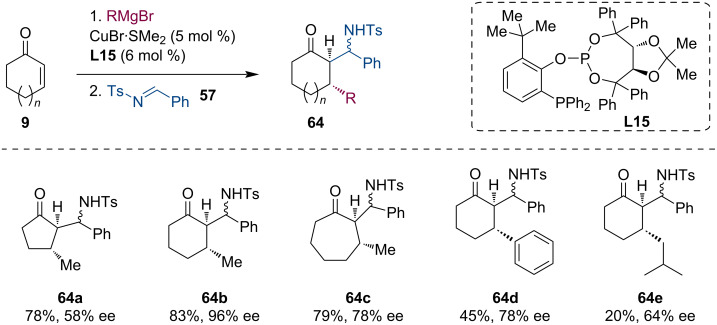
Tandem Grignard–ACA–Mannich using Taddol-based phosphine-phosphite ligands.

Within the framework of these domino reactions, we have mainly employed ferrocenyl phosphane ligands such as Taniaphos or Josiphos. In collaboration with Prof. Schmalz from Cologne University, we have also tested phosphite-phosphine ligands (e.g., **L15**) from their lab. The advantage of these ligands is that they can also promote the conjugate additions of aryl-based or branched Grignard reagents (Scheme 17) [49].

**Scheme 18:**
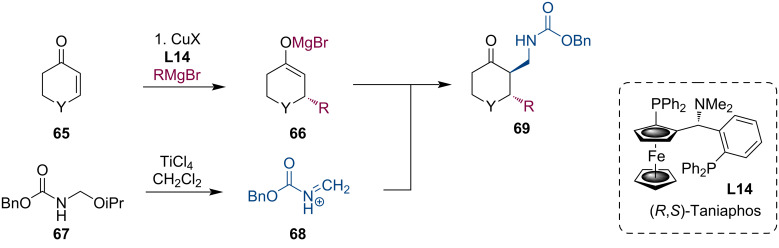
Tandem reaction of Mg enolates with aminomethylating reagents.

Further extending this methodology, we have investigated formaldehyde imine equivalents. These kinds of imines are not readily available, but they are highly important synthetic building blocks providing an aminomethyl moiety upon adding nucleophiles. Protected formaldehyde aminals are useful synthetic equivalents to formaldehyde imines. The imine functionality can be unmasked (**68**) in the reaction medium by Lewis acids such as TiCl_4_. The formed Mg enolates **66** readily react with the transient iminium species **68** to afford the corresponding aminomethylation products **69** (Scheme 18) [50]. As seen from Table 2, the diastereoselectivities were somewhat compromised compared to what one can expect from the reactions of cyclic enolates. This erosion was likely caused by Lewis acid-mediated epimerization.

**Table 2:** Domino aminomethylation of cyclic ketones with Grignard reagents.

RMgX	Yield	dr	ee (*trans*)	ee (*cis*)

MeMgBr	66	2:1	92	95
MeMgI	27	2.7:1	84	88
*n-*PentMgBr	63	2.4:1	92	60
iPentMgBr	34	2.2:1	92	60
cyclopentylMgBr	16	n.d.	74	74
HexMgBr	41	1.8:1	92	92

**Scheme 19:**
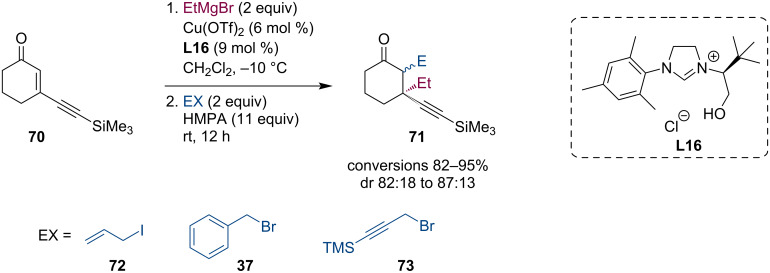
Tandem reaction composed of Grignard ACA to alkynyl enones.

Guénée et al. described the allylation, benzylation, and propargylation of magnesium enolates. These enolates were generated by a Cu-NHC-catalyzed conjugate addition of Grignard reagents to β-substituted cyclic enones (**70**) (Scheme 19) [51].

**Scheme 20:**
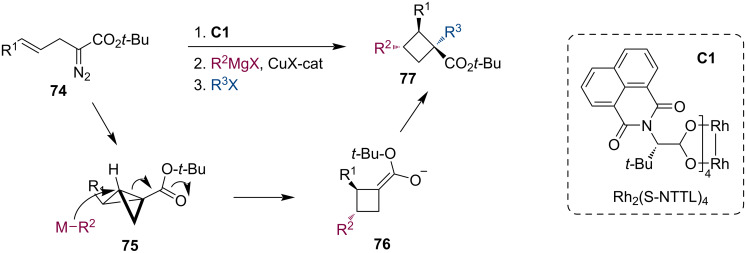
Rh/Cu-catalyzed tandem reaction of diazo enoates leading to cyclobutanes.

Fox and co-workers developed an intriguing synthesis of enantiomerically enriched cyclobutanes **77** [52]. Their strategy employed a three-component process in which *tert*-butyl (*E*)-2-diazo-5-arylpent-4-enoates **74** were treated with the chiral rhodium catalyst **C1** to provide enantiomerically enriched bicyclobutanes **75**. These highly strained compounds then participated in the Cu-catalyzed homoconjugate addition of Grignard reagents and subsequent enolate trapping to give densely functionalized cyclobutanes **77** with high diastereoselectivity (Scheme 20). The enolates were alkylated, allylated, benzylated, benzoylated, and thienylated.

**Scheme 21:**
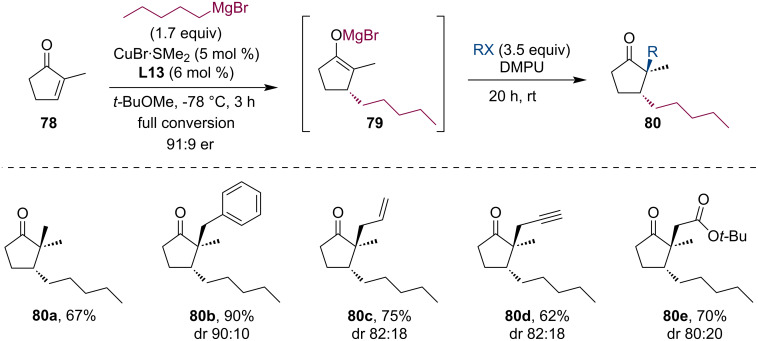
Tandem Grignard-ACA of cyclopentenones and alkylation of enolates.

Minnaard and co-workers developed a copper/Rev-Josiphos-catalyzed asymmetric conjugate addition of Grignard reagents to 2-methylcyclopentenone (**78**), which provided 2,3-disubstituted cyclopentanones in high yields and enantiomeric purities [53]. The one-pot alkylation reaction of the in situ formed magnesium enolate with alkylating reagents required the presence of 1,3-dimethyltetrahydropyrimidine-2(1*H*)-one (DMPU) (Scheme 21). Reactive alkylating reagents such as iodomethane, benzyl bromide, allyl iodide, propargyl bromide, or bromoacetate reacted well and afforded the products **80** in good yields.

**Scheme 22:**
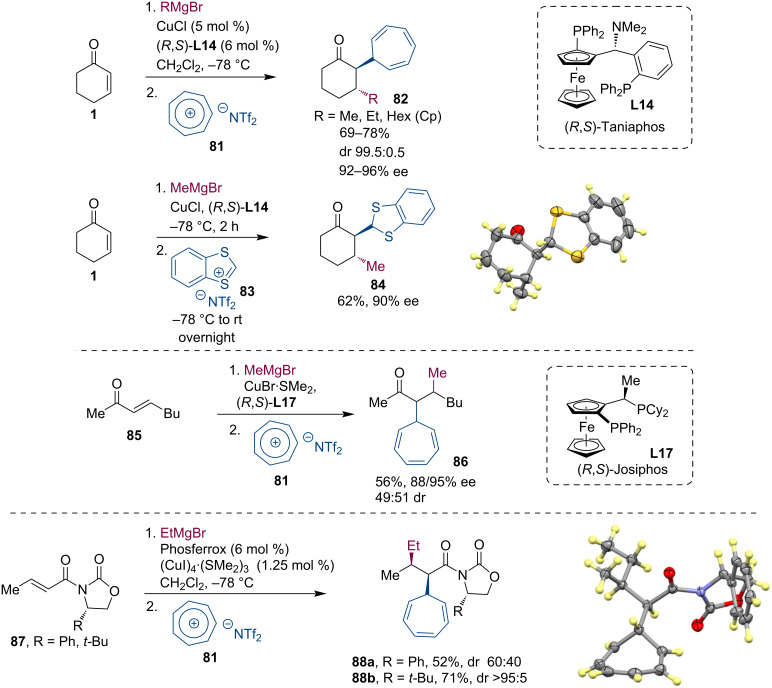
Tandem ACA of Grignard reagents followed by enolate trapping reaction with onium compounds.

In an attempt to expand the available electrophiles for reactions with metal enolates, we were inspired by the work of Cozzi and co-workers. They described reactions of organocatalytically generated enamines with stabilized carbenium ions [54–56]. These seminal results prompted us to push this question further and we asked whether metal enolates generated by conjugate additions would be compatible and react productively with suitable carbenium ions (Scheme 22). To investigate this question, we started our study with the well-known conjugate additions of Grignard reagents to cyclic and linear enones **1**, **85**, and **87**. At first, the addition of tropylium or benzodithiolium tetrafluoroborates were not highly productive because these onium compounds were not well soluble in typical solvents used for conjugate additions of organometallic reagents, e.g., Et_2_O, *t-*BuOMe or CH_2_Cl_2_. Therefore, we exchanged the BF_4_ anion in the onium compounds for the more lipophilic NTf_2_. This exchange led to more soluble onium compounds **81** and **83**, and consequently, also significantly improved the reaction with the metal enolates. As a result, the corresponding products were successfully isolated with tropylium and benzodithiolium cations [57]. The reaction worked well with Mg enolates generated from cyclic and linear enones **1** and **85** and enoyloxazolidinones **87**. Apart from the most robust tropylium and benzodithiolium cations, reactions were also possible with the dianisylmethylium cation. Interestingly, tritylium cations reacted only in the *para*-position of a phenyl ring, while flavylium triflate and 2,4,6-triphenylpyrylium tetrafluoroborate were not compatible with our reaction conditions.

**Scheme 23:**
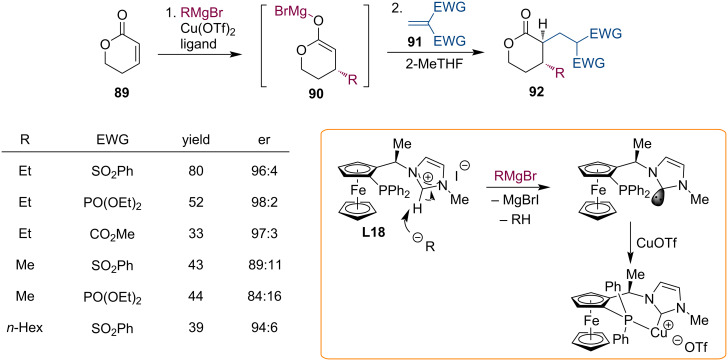
Mg enolates generated from unsaturated lactones in reaction with activated alkenes.

Heterodonor ferrocenyl phosphane–carbene ligands efficiently promote the conjugate addition of Grignard reagents to α,β-unsaturated lactones [58]. Building on this knowledge, we have investigated the domino reaction of the formed metal enolates with activated alkenes **91** [59]. Alkenes with two activating groups were needed for efficient enolate-trapping reactions, sulfone or phosphonate activating groups being the most suitable ones (Scheme 23).

**Scheme 24:**
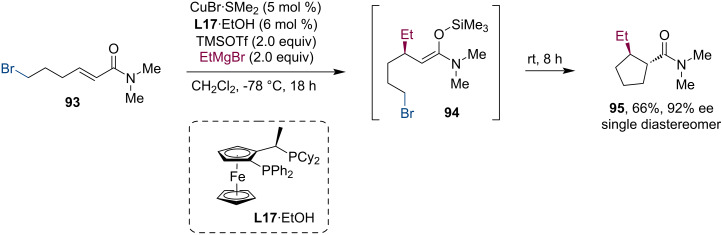
Lewis acid mediated ACA to amides and S_N_2 cyclization of a Br-appended enolate.

Harutyunyan and co-workers developed a Lewis acid-promoted conjugate addition to unreactive Michael acceptors such as amides or vinyl heterocycles [60]. Trimethylsilyl triflate or boron trifluoride-activated unsaturated amides underwent highly efficient and enantioselective addition of Grignard reagents. When this methodology was applied to a substrate with a pending bromo substituent (**93**), the formed enolate **94** underwent a spontaneous cyclization via an S_N_2 displacement (Scheme 24).

**Scheme 25:**
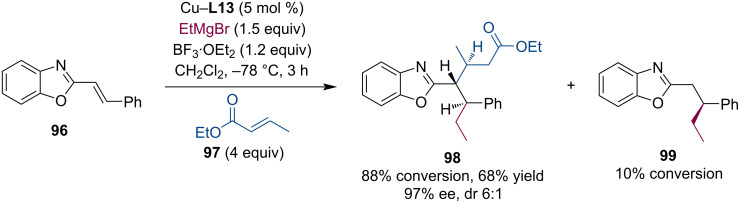
Trapping reactions of aza-enolates with Michael acceptors.

The Harutyunyan team showed that this methodology also applies to aza-enolates that are generated by the conjugate addition of Grignard reagents to alkenyl heteroarenes [61]. The aza-enolates were trapped with various Michael acceptors such as unsaturated ketones, esters, and amides (Scheme 25) [62]. The authors noted a strong substrate dependence of this process. The trapping reaction worked best with benzoxazole-derived substrate, while thiazole was also possible. Among electrophilic reagents, unsaturated esters worked best.

**Scheme 26:**
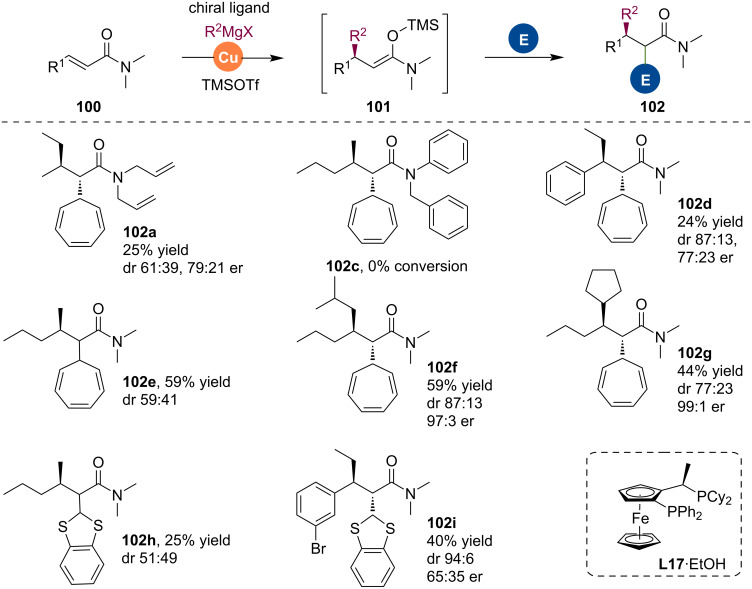
Si enolates generated by TMSOTf-mediated ACA of Grignard reagents and enolate trapping reaction with onium compounds.

In collaboration with the Harutyunyan group, we have further explored the possibilities of chiral enolate trapping which were obtained by asymmetric conjugate addition of organometallic reagents. We intended to employ the Lewis acid-mediated generation of magnesium enolates in the trapping reactions with carbocations. Indeed, unsaturated amides, alkenyl heterocycles, or even unsaturated carboxylic acids successfully participated in this process affording structurally interesting products (Scheme 26) [63].

**Scheme 27:**
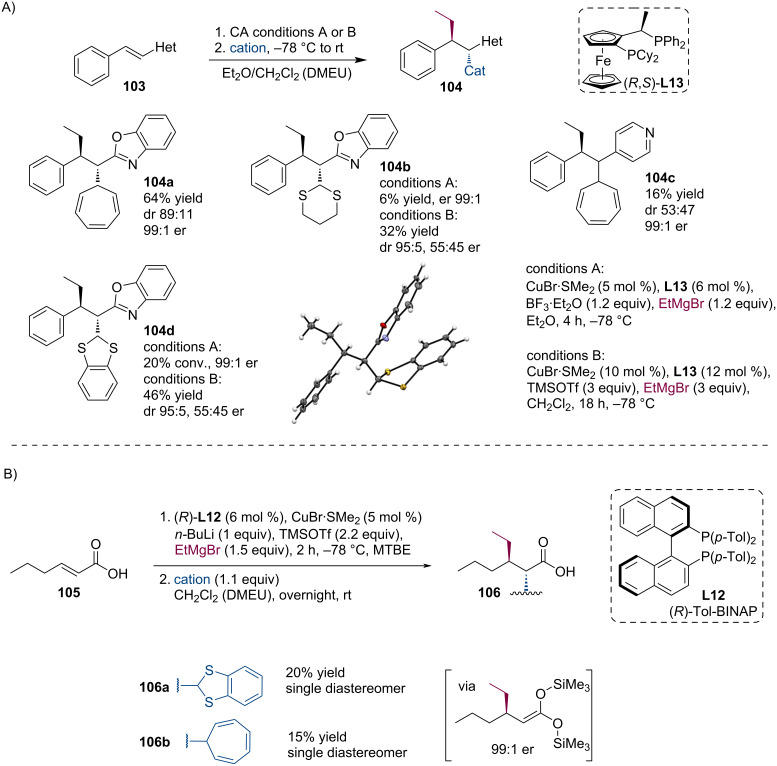
Trapping reactions of enolates generated from alkenyl heterocycles (A) and carboxylic acids (B) with onium compounds.

Apart from unsaturated amides, the trapping worked also with alkenylheterocycles **103**. Interestingly, the corresponding aza-enolates could be generated by two sets of experimental conditions where ACA was promoted either by BF_3_·OEt_2_ or TMSOTf (Scheme 27A). Based on the recent Harutyunyan discovery of ACA to unsaturated carboxylic acids [64], we have attempted a similar trapping reaction here as well. Gratifyingly, the corresponding trapping products **106** could be isolated with tropylium and benzodithiolium cations (Scheme 27B).

**Scheme 28:**
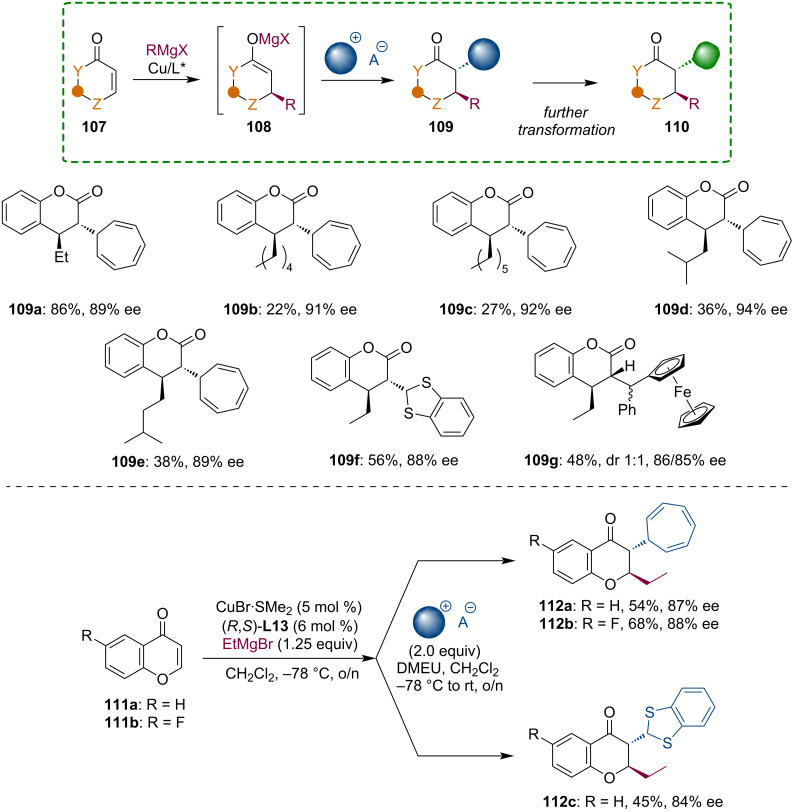
Reactions of heterocyclic Mg enolates with onium compounds.

We have continued our exploration of enolate reactions with carbocations by studying the trapping of heterocyclic enolates **108** generated from coumarin and chromone [65]. The high enantio- and diastereoselectivity of these transformations were ensured by a Josiphos-type ferrocene ligand. The reaction of chiral metal enolates with onium compounds enabled the installation of structurally attractive substituents on the chromenone or piperidinone core (Scheme 28).

**Scheme 29:**
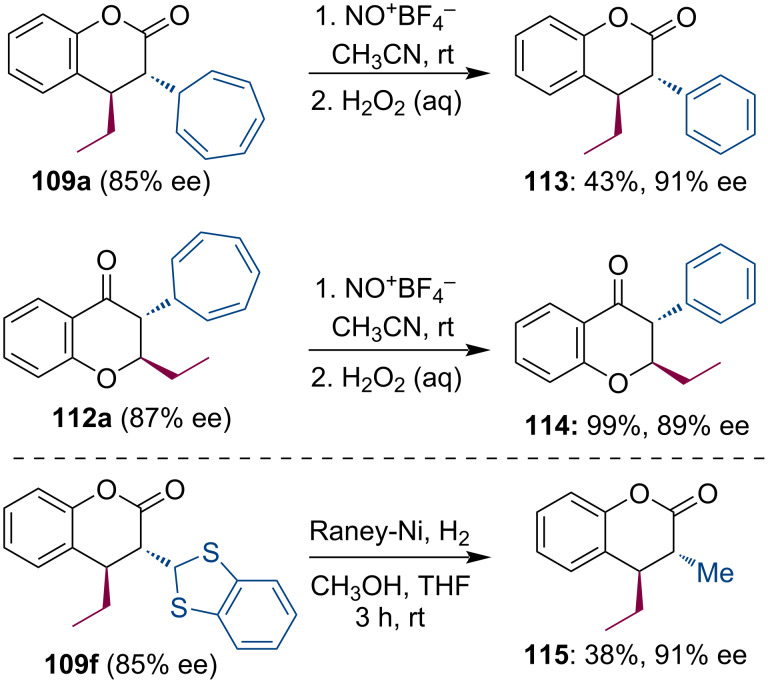
Synthetic transformations of cycloheptatrienyl and benzodithiolyl substituents.

Furthermore, cycloheptatrienyl and benzodithiolyl substituents can be further modified, thus, expanding the synthetic possibilities of this methodology. The cycloheptatrienyl substituent allows oxidative ring contraction to form a phenyl ring, which is otherwise not easy to introduce into the C-2 position of carbonyl compounds. Finally, the benzodithiolyl group can be reduced into a methyl group (Scheme 29).

### Conjugate additions with trialkylaluminum reagents

**Scheme 30:**
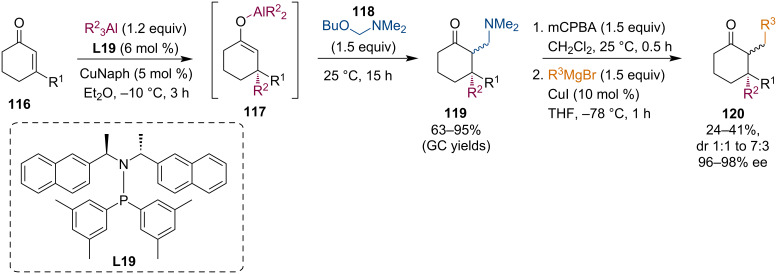
Aminomethylation of Al enolates generated by ACA of trialkylaluminum reagents.

Conjugate additions of trialkylaluminum reagents are somewhat less populated as a basis for generating and trapping of reactive metal enolates. The conjugate addition of R_3_Al to cyclic enones catalyzed by a combination of copper(II) naphthenate (CuNaph) and SimplePhos ligand **L19** led to the corresponding aluminum enolates. Alexakis and co-workers used these enolates in a Mannich-type reaction with the α-aminoether **118**. This reagent released an iminium ion into the reaction medium that reacted with the Al enolate **117** [66]. Furthermore, the Mannich adduct was then reacted with Grignard reagents that replaced the dimethylamino group (Scheme 30).

**Scheme 31:**
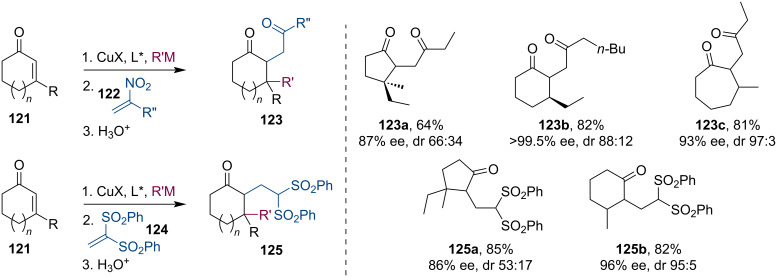
Trapping reactions of enolates with activated alkenes.

Alexakis and co-workers also investigated the trapping of metal enolates by Michael reactions with nitroalkenes **122** and disulfonyl ethylenes **124** (Scheme 31) [67]. The conjugate additions of dialkylzinc, Grignard, and trialkylaluminum reagents to cyclic enones **121** were realized using previously established chiral phosphoramidite, carbene or ferrocene ligands. All types of metal enolates generated via these processes were able to react with Michael acceptors and afforded the corresponding products in good yields.

**Scheme 32:**
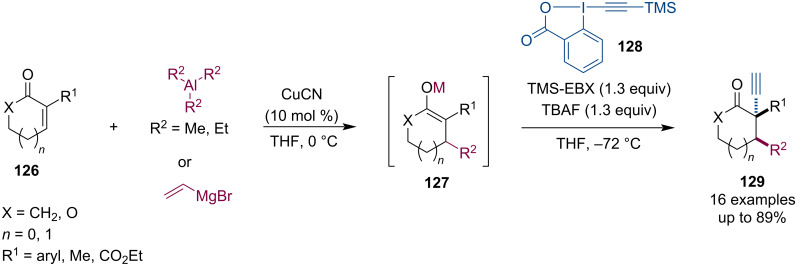
Alkynylation of racemic aluminum or magnesium enolates.

The alkynylation of enolates generated by conjugate addition was described by Teodoro and Silva (Scheme 32) [68]. Even though the conjugate addition of trialkylaluminum or Grignard reagents was realized only in an achiral manner, this work merits discussion here. Aluminum and magnesium enolates were alkynylated with ethynylbenziodoxolone (EBX). This diastereoselective electrophilic alkynylation afforded the corresponding α-alkynylketones **129** in good yields.

### Conjugate additions with organozirconium reagents

**Scheme 33:**
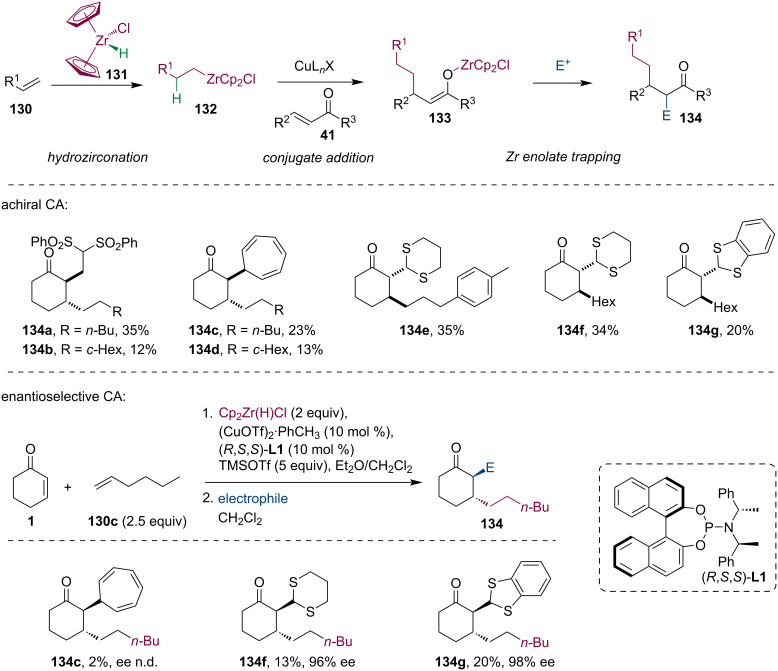
Trapping reactions of Zr enolates generated by Cu-ACA of organozirconium reagents.

The hydrozirconation of alkenes and alkynes generates mild organozirconium compounds that can be used in various transformations. Fletcher and co-workers developed the utilization of organozirconium reagents in Cu-catalyzed conjugate additions and allylic substitutions [15]. Given these developments, we posed the question of how would Zr enolates **133**, formed by the corresponding conjugate addition, would react with highly reactive electrophiles. With typical electrophiles such as benzaldehyde or nitrostyrene no enolate trapping was observed, however, we isolated trapping products with onium compounds having tropylium, benzodithiolium, and 1,3-dithian-2-ylium cations. The zirconium enolate also reacted with a highly activated alkene (Scheme 33) [69]. A comparison with related Mg and Si enolates revealed a lower reactivity of zirconium enolates, presumably, due to the considerable steric hindrance caused by bulky ligands around the Zr center.

**Scheme 34:**
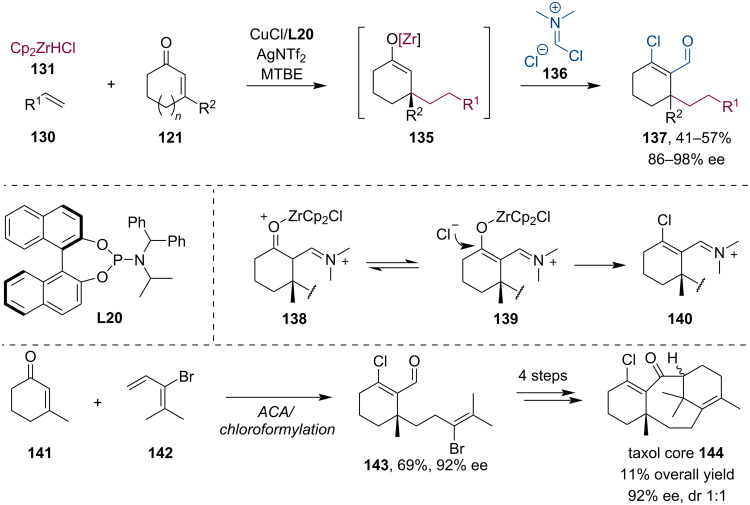
Chloromethylation of Zr enolates using the Vilsmeier–Haack reagent.

Fletcher investigated the formylation of zirconium enolates with the Vilsmeier–Haack reagent [70]. Interestingly, the reaction afforded chloroformylation rather than simple formylation products. The methodology was later exploited in the expedient synthesis of the Taxol core (Scheme 34) [71].

### Tandem conjugate borylations and silylations

**Scheme 35:**
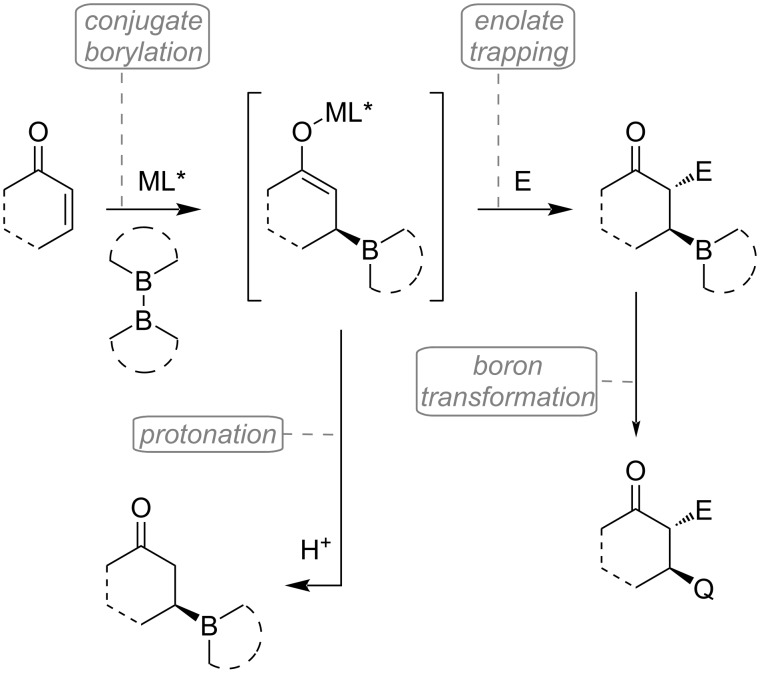
Tandem conjugate borylation with subsequent protonation or enolate trapping by an electrophile.

Chiral organoboron compounds are well-known synthetic building blocks with diverse possibilities for subsequent derivatization (e.g., oxidation, transformation to potassium trifluoroborate salt, hydrolysis, C–C cross-coupling, base-mediated elimination, radical C–B cleavage) [72]. Therefore, enantioenriched boronates are commonly applied intermediates in organometallic, medicinal, and other fields of chemistry. At the same time, some organoboronic acid derivatives have been found to exhibit potent biological activities, which has led to the development of several FDA-approved drug molecules [73–74]. Following the seminal works of Hosomi [75] and Miyaura [76], the synthesis of β-boron-substituted carbonyl compounds by conjugate addition of boron species to activated alkenes has matured into a well-developed strategy (Scheme 35).

**Scheme 36:**
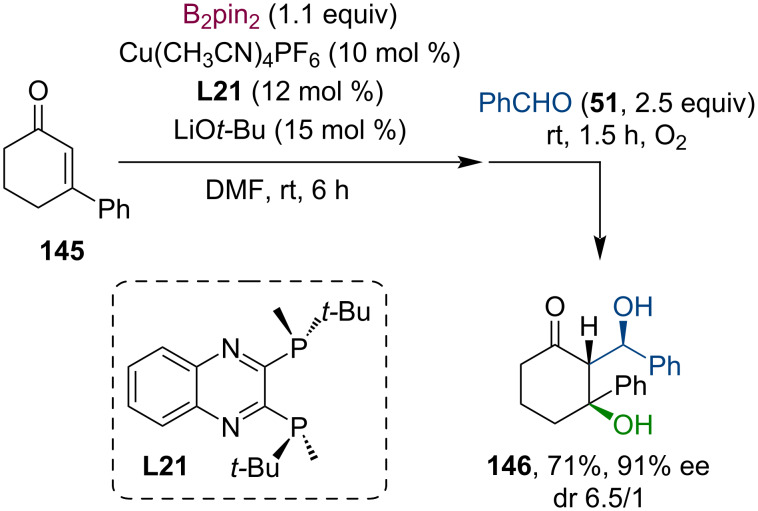
Tandem conjugate borylation/aldol reaction of cyclohexenones.

Despite its ability to build complex structures, the conjugate borylation with subsequent enolate trapping has rarely been applied in the last decade. These few examples are mostly limited to aldol reactions. In 2009, Shibasaki and co-workers explored the copper-catalyzed asymmetric conjugate borylation of β-substituted cyclic enones using chiral bisphosphine ligand **L21** [77]. Other than the oxidation and hydrolysis of the produced enantiomerically enriched tertiary boronates, in one example, they have demonstrated the utilization of the enolate intermediate in a cascade sequence, including borylation, aldol reaction, and finally oxidation (Scheme 36). The product **146** containing three consecutive stereocenters was obtained in a dr of 6.5 to 1 and with good yield and enantioselectivity.

**Scheme 37:**
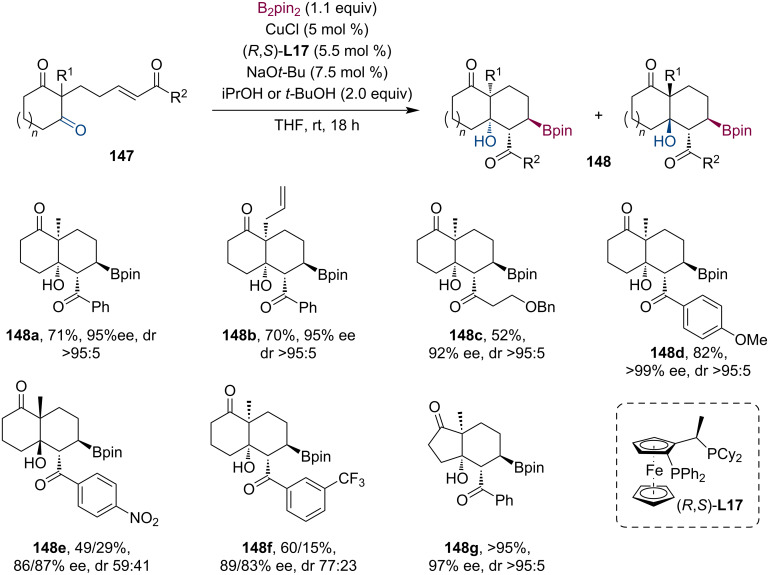
Selected examples for the tandem asymmetric borylation/intramolecular aldol reaction; synthesis of functionalized bicyclic compounds.

Lam and co-workers described a highly enantioselective tandem borylation/intramolecular aldol cyclization procedure (Scheme 37) [78]. The desymmetrization process of cyclic diones **147** gave the densely functionalized bicyclic products **148** with four contiguous stereocenters usually in a highly diastereoselective fashion. Presumably, the difference in diastereocontrol originates from the preferred *E*/*Z* enolate geometry during the transition state. Interestingly, using *t-*BuOH instead of iPrOH resulted in exceptionally better results for some substrates with different ring sizes.

**Scheme 38:**
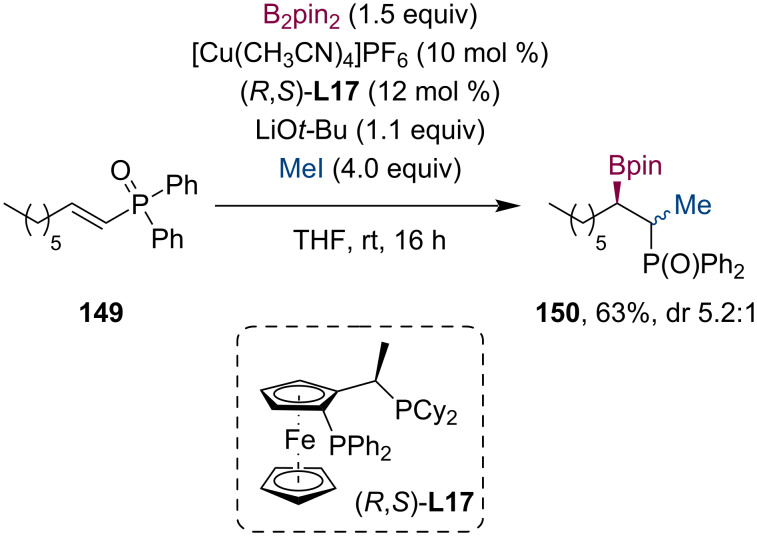
Cu-catalyzed tandem methylborylation of α,β-unsaturated phosphine oxide in the presence of (*R*,*S*_p_)-Josiphos ligand (**L17**).

In 2015, the group of Feringa investigated the copper-catalyzed conjugate borylation of α,β-unsaturated phosphine oxides **149** [79]. Their work also included an example of the consecutive trapping of the enolate by MeI (Scheme 38). Using the (*R*,*S*_p_)-Josiphos ligand (**L17**), the product of the tandem reaction (**150**) was gained in 63% yield (dr 5.2:1).

**Scheme 39:**
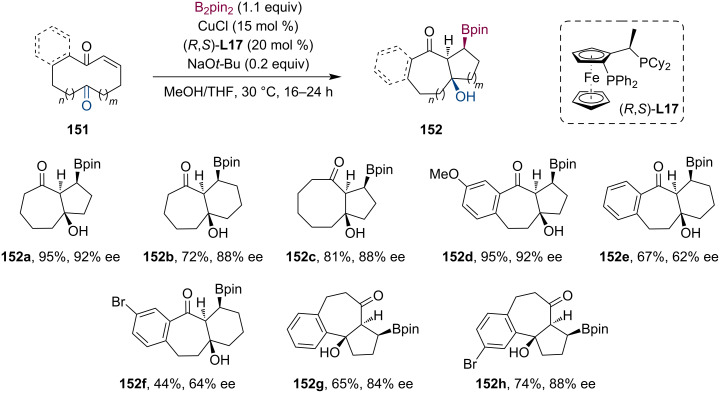
Cu-catalyzed tandem transannular conjugated borylation/aldol cyclization of macrocycles containing an α,β-unsaturated ketone and an additional ketone moiety.

At the beginning of the new decade, Fernández et al. presented a Cu-catalyzed tandem borylation/transannular aldol cyclization using decane and undecane macrocyclic substrates **151** (Scheme 39) [80]. This methodology enabled the synthesis of complex bicyclic scaffolds in a completely diastereoselective and straightforward manner. Based on their NMR experiments (H_α_ coupling constants ≈ 6.4–8.6 Hz), the high level of diastereocontrol can be associated with the preferred *Z*-configuration of the cyclic copper enolate intermediate. In the presence of the chiral ligand (*R*,*S*)-**L17**, the tandem reaction was accomplished in a highly enantioselective way (ee up to 92%).

**Scheme 40:**
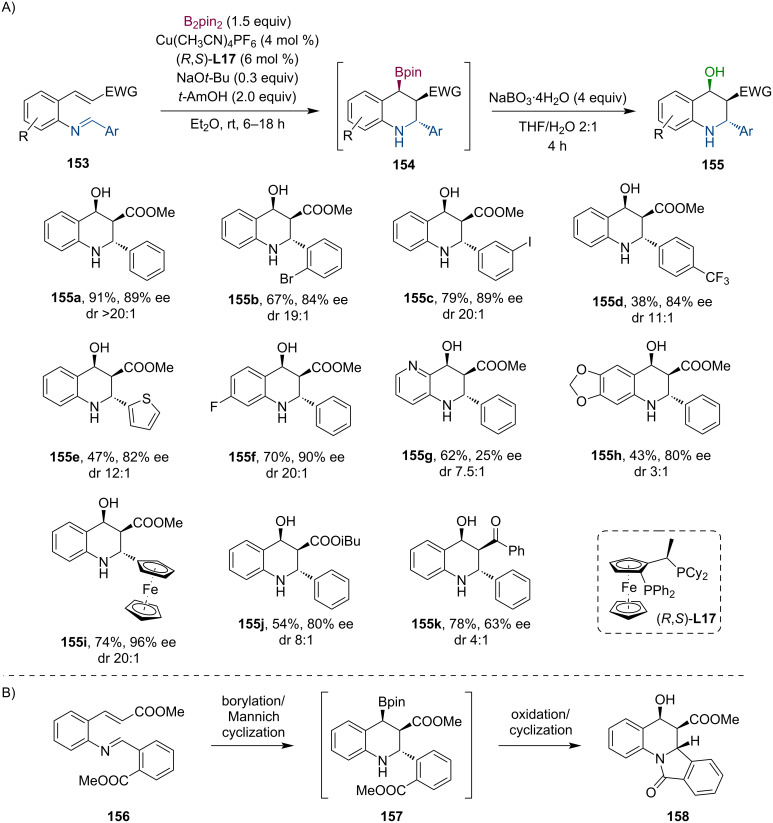
Stereoselective tandem conjugate borylation/Mannich cyclization: selected examples (A) and a multi-electrophile cascade reaction furnishing a complex tetracyclic scaffold (B).

In the same year, Lautens and co-workers introduced a novel methodology for preparing enantioenriched N-heterocycles utilizing a Cu-catalyzed tandem conjugate borylation/Mannich cyclization sequence (Scheme 40A) [81]. The procedure was found to be generally relevant as several structurally different Michael acceptors were successfully applied. Their work also included a 3 mmol scale-up (62%, 87% ee, dr >20:1) and various derivatizations of the Mannich products. Furthermore, they have also attempted a multi-electrophile cascade reaction, which harnesses the nucleophilic nature of the secondary amine **157** generated in the cyclization step (Scheme 40B). Consequently, the complex tetracyclic compound **158** was produced in 47% yield and with good stereoselectivity (84% ee, dr 7.5:1).

**Scheme 41:**
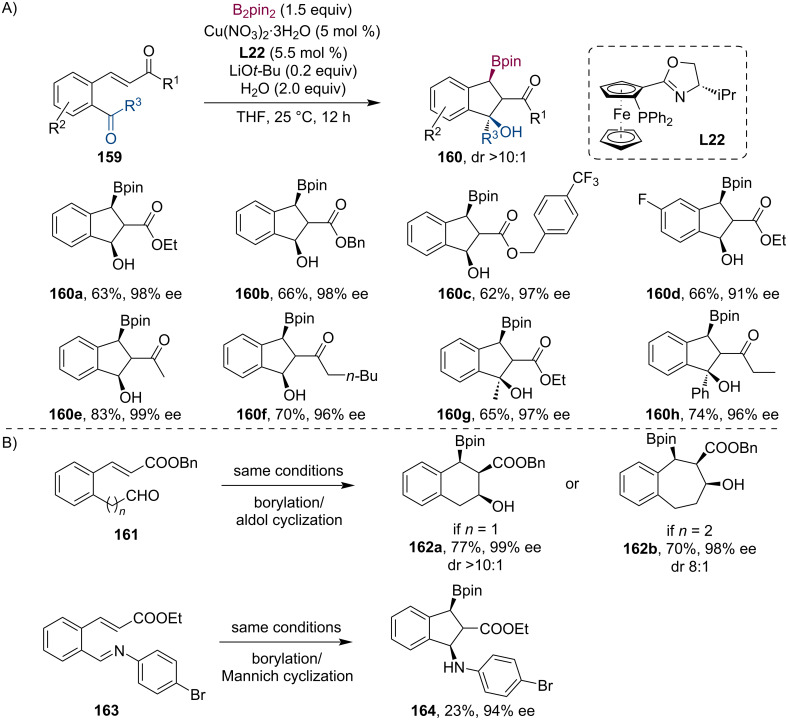
Some examples of Cu-catalyzed asymmetric tandem borylation/aldol cyclization (A). Application to different ring sizes and combinations with Mannich reaction (B).

At about the same time, comparable results were reported by the group of Zhang [82]. Their Cu-catalyzed cascade borylation/aldol cyclization methodology provides rapid access to various indane derivatives **160** (Scheme 41A) with good yields and excellent chemo-, and stereoselectivities. Next, they successfully extended this process to 6-, and 7-membered benzocyclic compounds and the corresponding boronates **162** were isolated with equally good yields and excellent stereoselectivities (98–99% ee) (Scheme 41B). Surprisingly, when the aldehyde was exchanged with imine **163**, the Mannich product **164** was gained only in 23% yield, however, with excellent enantioselectivity of 94%.

**Scheme 42:**
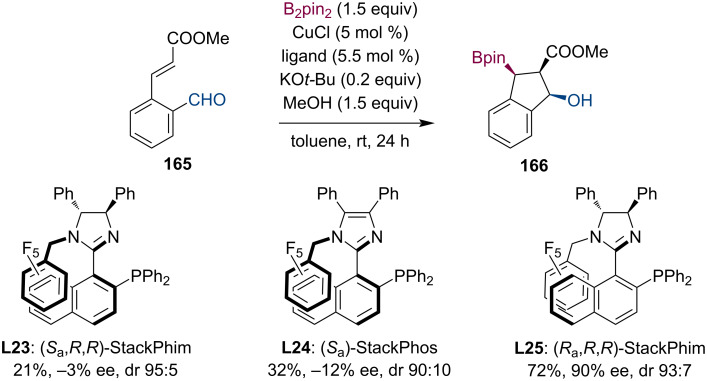
Atropisomeric P,N-ligands used in tandem conjugate borylation/aldol cyclization sequence.

In the following year, Aponick et al. systematically modified atropisomeric C1-symmetric stack ligands to identify suitable catalytic systems for a highly enantioselective synthesis of organoboranes (Scheme 42) [83]. Their best attempt to realize a tandem borylation/aldol cyclization reaction resulted in 72% yield, 90% ee, and a diastereomeric ratio of 93:7 using ligand **L25**.

**Scheme 43:**
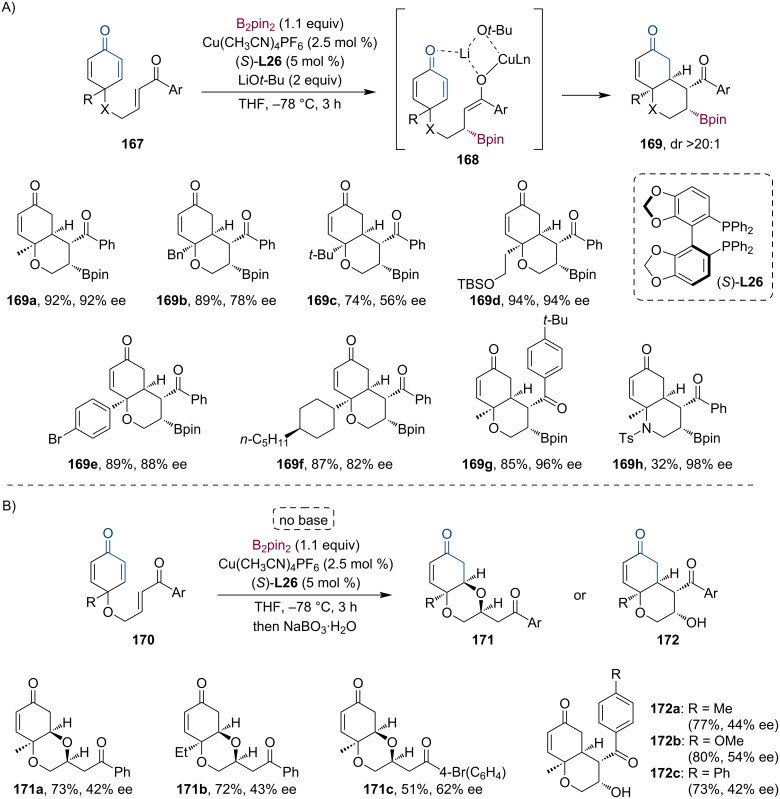
Selected examples for the enantioselective Cu-catalyzed borylation/intramolecular Michael addition (A). Without the base, the reaction proceeds through a tandem borylation/oxidation/oxa-Michael sequence (B).

Recently, Chegondi and co-workers have demonstrated an enantioselective Cu-catalyzed tandem borylation/Michael addition reaction of aryl enones **167** and **170** to cyclohexadienones in an intramolecular fashion (Scheme 43A) [84]. The 1,4-conjugate borylation is followed by a desymmetrization step during which the chiral enolate attacks (*Si*-face) the prochiral cyclohexadienone ring via a chair-like transition state. The reaction requires an excess amount of base, resulting in the formation of a more favorable lithium enolate. Subsequent oxidation of the boronates gave the corresponding alcohols without a significant change in yield or selectivity. Interestingly, in the absence of the base, the reaction led to fused dioxane derivatives (Scheme 43B). This can be explained by a borylation/oxidation/oxa-Michael tandem sequence instead of the *C*-Michael addition. The role of the base was thoroughly examined using DFT calculations. Other than the broad substrate scope, the synthetic utility of this method was demonstrated by a scale-up reaction (3.73 mmol scale, 87% yield, 88% ee), and by several different transformations of the tandem products.

**Scheme 44:**
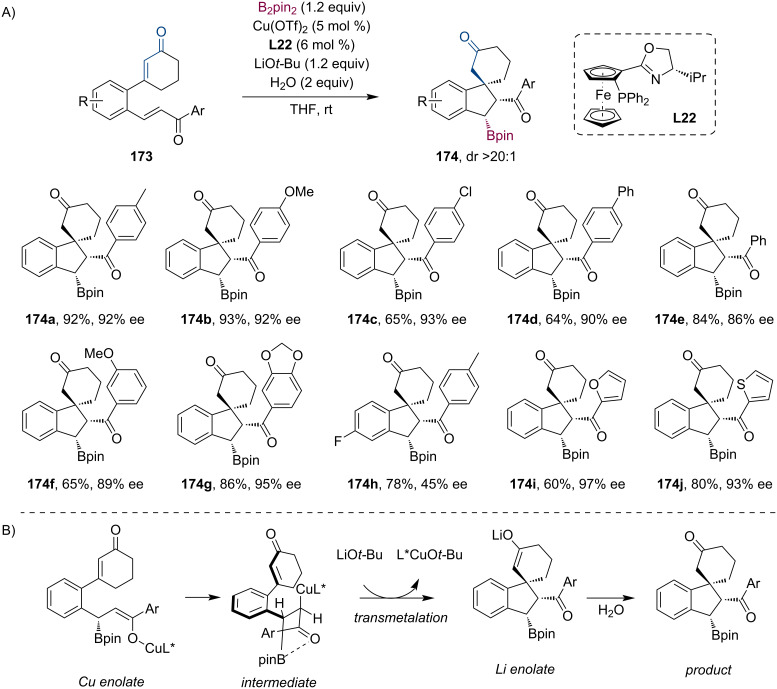
Selected examples for the preparation of enantioenriched spiroindanes using a Cu-catalyzed tandem conjugate borylation/Michael addition sequence (A). Role of the stoichiometric base (B).

Similarly, Ghorai et al. studied a Cu-catalyzed cascade borylation/Michael addition sequence leading to enantioenriched spiroindane boronates **174** (Scheme 44A) [85]. The reaction showed good functional group tolerance. Further derivatization, as well as scale-up (1 mmol) of the reaction were successfully performed (72% yield, 89% ee, dr >20:1). Based on their control experiments and literature mechanistic studies (Chegondi et al.) [84], the role of the base (LiO*t-*Bu) was considered. Following the Cu-catalyzed conjugate addition of B_2_pin_2_, the Michael cyclization is facilitated by the transmetalation of stoichiometric Li base with the Cu enolate (Scheme 44B). In the end, protonation of the Li enolate affords the spiroindane boronate.

**Scheme 45:**
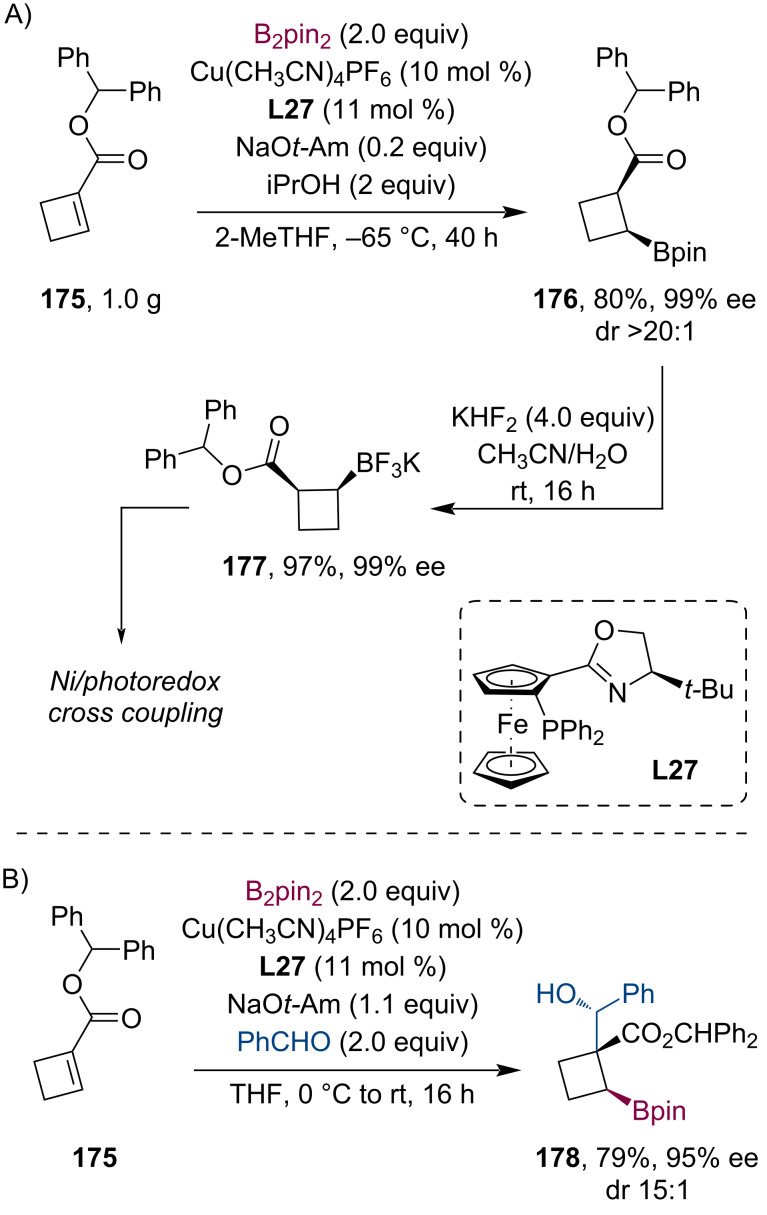
Enantioselective conjugate borylation of cyclobutene-1-carboxylic acid diphenylmethyl ester **175** with subsequent enolate trapping using benzaldehyde.

Due to their paramount role in the fields of bioactive natural products and medicinal chemistry, there is a growing interest in enantioenriched cyclobutanes. Recently, the group of Hall was engaged in developing enantioselective methods for the synthesis of cyclobutylboronates which could serve as important building blocks [86]. Utilizing high-throughput (HTS) chiral ligand screening, they have presented the highly asymmetric conjugate borylation of disubstituted cyclobutenones. Next, they thoroughly studied the stereoselective conjugate borylation of cyclobutene 1-carboxyester **175** (Scheme 45A) [87]. As a result, the *cis*-β-boronyl cyclobutylcarboxyester **176** was prepared on a gram scale with 80% yield and an excellent 99% ee (dr >20:1). Subsequent transformation to the corresponding trifluoroborate salt **177** resulted in a highly beneficial scaffold which was successfully involved in diastereoselective Ni/photoredox dual-catalyzed cross-coupling reactions. Furthermore, rather than the stereoselective protonation, they have also demonstrated the successful trapping of the Cu enolate with benzaldehyde (Scheme 45B). This tandem conjugate borylation/aldol reaction gave the aldol product **178** in 79% yield and an exceptional 95% ee (dr 15:1).

**Scheme 46:**
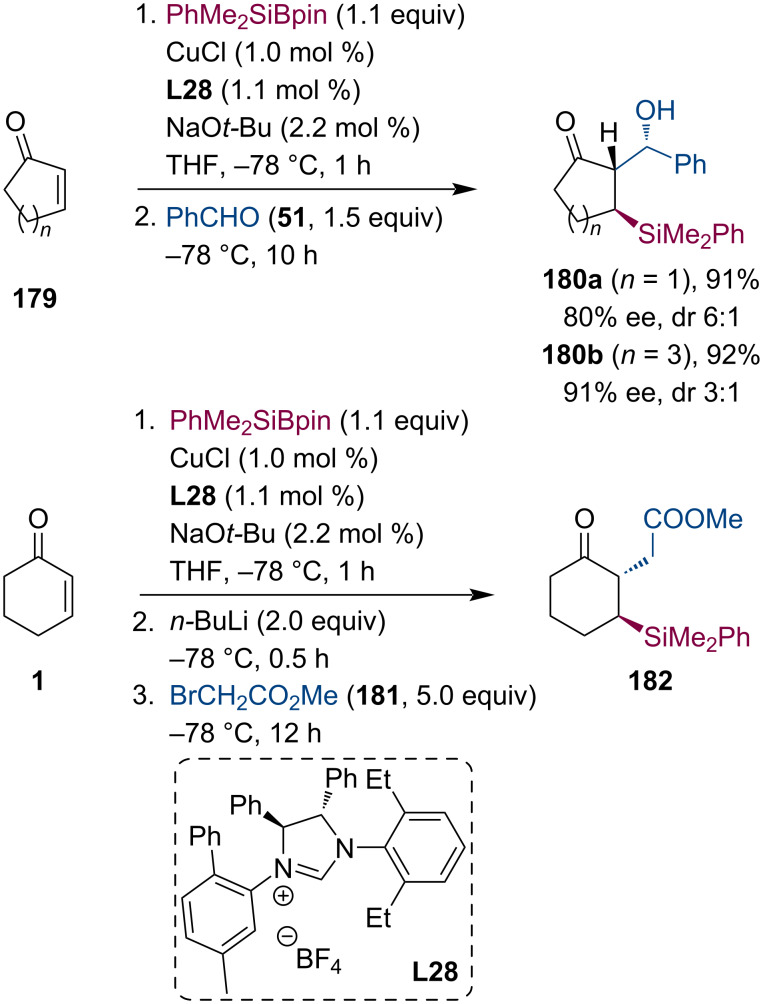
Cu-catalyzed enantioselective tandem conjugate silylation of α,β-unsaturated ketones with subsequent enolate trapping.

Similarly to conjugate borylation, silyl functional groups can be also introduced into activated alkenes. Furthermore, the additional transformation of the silyl motif might be similar or even complementary to boronates (e.g., sensitivity to organometallic reagents). In 2010, the Hoveyda group accomplished the NHC–Cu-catalyzed enantioselective conjugate addition of PhMe_2_Si-Bpin to α,β-unsaturated enones [88]. Additionally, they have also presented the successful trapping of the Cu enolate intermediate with benzaldehyde (**51**) and methyl bromoacetate (**181**) (Scheme 46).

**Scheme 47:**
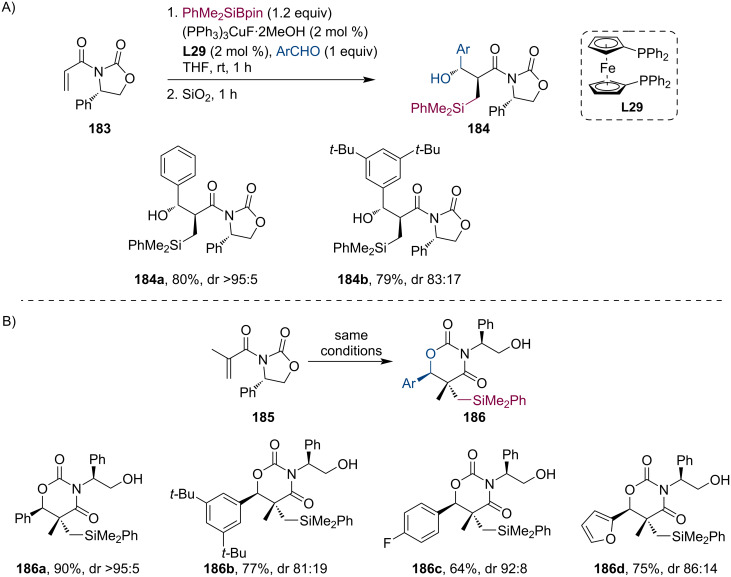
Cu-catalyzed enantioselective tandem conjugate silylation of α,β-unsaturated ketones with subsequent enolate trapping.

At about the same time, Riant and co-workers investigated the chiral auxiliary-assisted Cu-catalyzed tandem silylation/aldol reaction between enoyloxazolidinones and different aromatic aldehydes [89]. In the case of acryloyloxazolidinone **183**, the reaction gave the expected aldol product **184** in good yields and diastereomeric ratios with a preference toward the *syn*-adduct (Scheme 47A). Interestingly, when they used methacryloyloxazolidinone **185** as a Michael acceptor, the X-ray analysis of the product showed a rearranged structure (Scheme 47B). The authors concluded that the new structure **186** is formed by intramolecular ring opening of the oxazolidine unit initiated by the hydroxy group either following the aldol condensation or during the reaction workup.

**Scheme 48:**
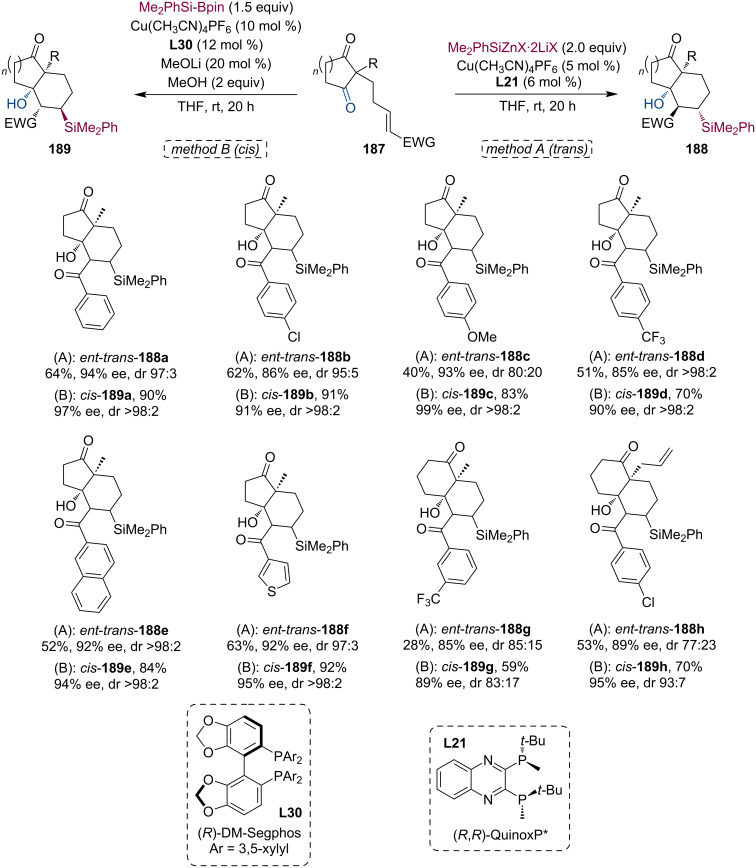
Cu-catalyzed tandem conjugate silylation/aldol condensation. The diastereoselectivity is controlled by the silicon nucleophile.

In 2021, Zhang and Oestreich presented a Cu-catalyzed tandem conjugate silylation/aldol cyclization sequence where the diastereoselectivity of the reaction is determined by the Si nucleophile used [90]. Using Me_2_PhSiZnX·2LiX in combination with ligand **L21** leads to the *trans* adduct, while Me_2_PhSiBpin together with **L30** provides the *cis* product. Consequently, the authors have successfully synthesized a broad range of bicyclic structures with excellent enantio- and diastereocontrol (Scheme 48). The thermodynamically driven *cis*-to-*trans* isomerization is also available by a retro-aldol–aldol procedure facilitated by a strong base (NaOH or Me_2_PhSiZnX·2LiX). Additionally, further derivatization is possible through the oxidation of the silyl motif to alcohol or the dehydration of the aldol adduct.

### Other tandem conjugate addition/enolate-trapping reactions

**Scheme 49:**
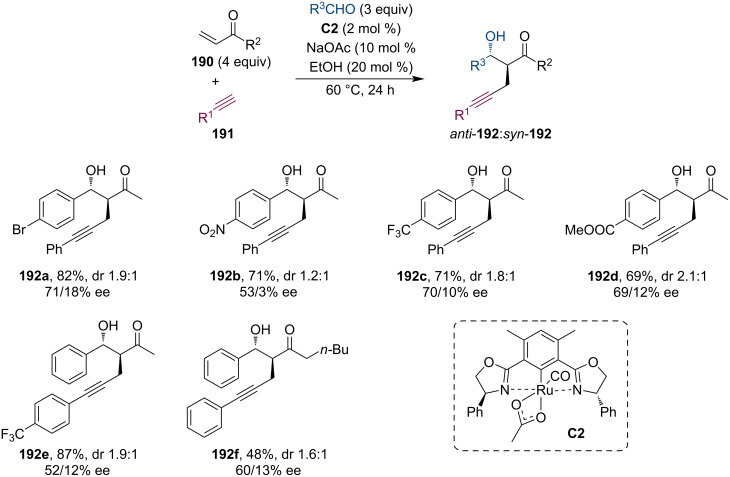
Chiral Ru-catalyzed three-component coupling reaction.

In 2016, Nishiyama and co-workers have studied a three-component coupling reaction of alkynes, enones, and aldehydes via direct conjugate alkynylation and consecutive aldol addition (Scheme 49) [91]. The chiral ruthenium complex **C2** (Phebox-type)-catalyzed procedure delivered β-hydroxyketone derivatives **192** having α-propargyl groups in good yields, however, only with low diastereoselectivities (up to 3:1). While the *syn*-diastereomers had low ee values, the *trans*-products showed better enantioselectivities (up to 78%). Their control experiments suggested that the Ru enolate, formed by the conjugate addition of the alkyne to the enone, plays a significant role in the following aldol reaction.

**Scheme 50:**
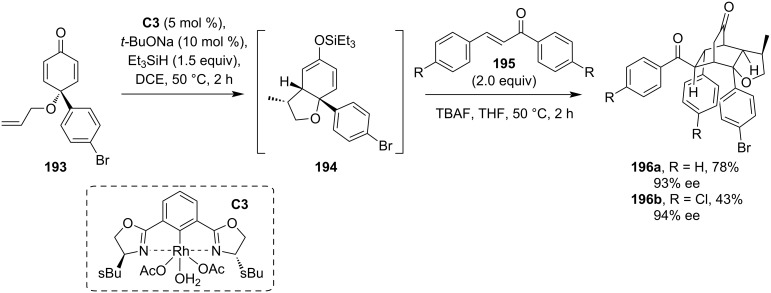
Rh-Phebox complex-catalyzed reductive cyclization and subsequent reaction with Michael acceptors through the silyl enol ether.

Later, Tian et al. have also employed a Phebox-based rhodium complex (**C3**) to catalyze the tandem conjugate addition of a terminal alkene followed by reacting the bicyclic dienol silyl ether intermediate with Michael acceptors in a one-pot procedure (Scheme 50) [92]. The bridged cyclic products **196a**,**b**, formed by a double Michael addition sequence, were isolated in moderate to good yields and with high enantiopurities.

**Scheme 51:**
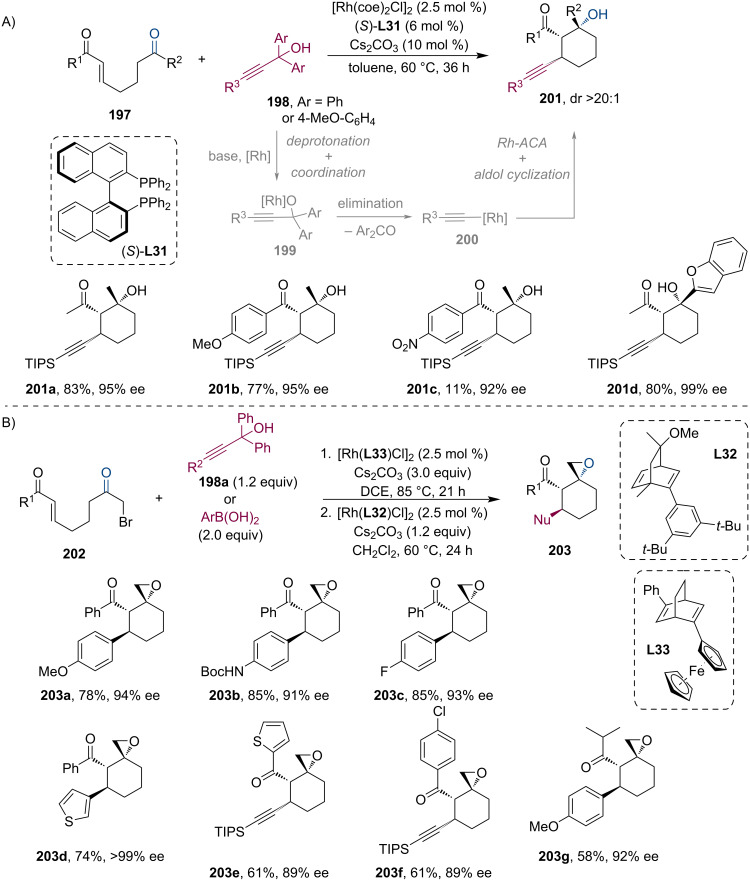
Rh-catalyzed tandem asymmetric conjugate alkynylation/aldol reaction (A) and subsequent spiro-cyclization (B).

Continuing with other Rh-based catalysts, the group of Lautens has also studied the stereoselective conjugate addition of alkynyl species to α,β-unsaturated ketones with subsequent trapping of the metal enolate by aldol cyclization (Scheme 51A) [93]. The reaction starts with the coordination of the Rh catalyst to the propargyl alcohol **198**. In the presence of a base, the rhodium–alkynyl reagent is generated with the concomitant extrusion of benzophenone. Finally, the alkynylation of the enone is followed by the cyclization step which yields the α-propargyl-β-hydroxyketones **201** in good yields and excellent diastereo- and enantiopurities. Soon after, the Lautens group has further extended their methodology to the synthesis of spirooxirane derivatives **203** by implementing a spiro-cyclization step following the aldol reaction (Scheme 51B) [94]. Giving only a single diastereomer with good enantioselectivity using a Rh/bicyclo[2.2.2]octane-2,5-diene (bod) complex, a broad variety of spiro compounds were isolated in good to excellent yields. The authors have also shown that this skeleton provides a great opportunity to prepare complex molecules by further transformations.

**Scheme 52:**
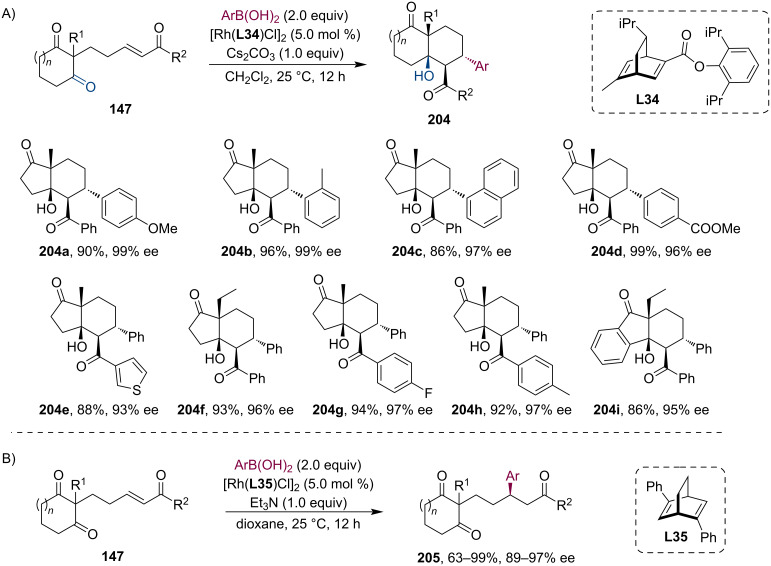
Rh-bod complex-catalyzed tandem asymmetric conjugate arylation/intramolecular aldol addition (A). Simple asymmetric arylation without the subsequent cyclization in the presence of triethylamine (B).

Similarly, Huang et al. have recently published their work on the Rh-bod complex-catalyzed highly stereoselective tandem arylation/aldol cyclization [95]. The conjugate addition of arylboronic acids to acyclic α,β-unsaturated ketones **147** with sequential intramolecular addition of the enolate to the cyclic dione moiety resulted in various bicyclic compounds with quantitative diastereoselection and excellent yields and enantioselectivities up to 99% (Scheme 52A). Interestingly, when the authors exchanged the Cs_2_CO_3_ base to Et_3_N, the hydroarylated derivative **205** was isolated as the main product (Scheme 52B). Thus far, the direct hydroarylation of such enone-dione substrates was unprecedented, presumably, due to the preferred metal-catalyzed aldol cyclization. Their protocol was further verified by a wide substrate scope. Additionally, the reaction showed high functional group tolerance with excellent stereoselectivities.

**Scheme 53:**
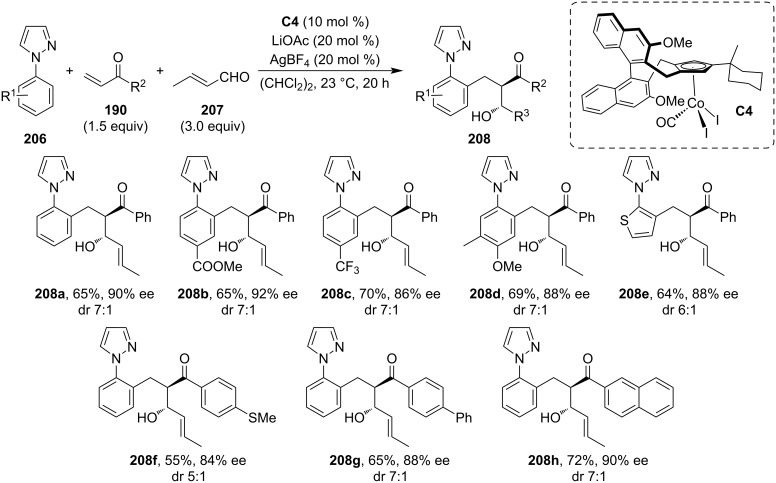
Co-catalyzed C–H-bond activation/asymmetric conjugate addition/aldol reaction.

In 2016, Ellman and co-workers demonstrated a Rh- or Co-catalyzed highly diastereoselective tandem C–H bond addition/aldol reaction sequence [96–97]. The C–H activation was promoted by pyridine, pyrazole, or imine directing groups, while the aldol addition step was performed either in a two-component (intramolecular aldol) or a three-component (intermolecular aldol) arrangement. The enantioselective implementation of this methodology was realized by Herraiz and Cramer in 2021 (Scheme 53) [98]. The reaction sequence is initiated by the C–H activation of aryl pyrazoles, followed by the asymmetric conjugate addition to the Michael acceptor. Then, the formed cobalt enolate participates in the intermolecular aldol reaction with an aldehyde **207**. The stereochemistry of this tandem procedure is controlled by the chiral Co(III) complex **C4** bearing binaphthyl-derived Cpx ligands. The authors have successfully isolated a broad scope of β-hydroxyketones **208** in good yields and high enantioselectivities. Although only a moderate diastereomeric ratio was achieved, their ligand screening showed that properly tuning the ligand structure can significantly affect the diastereomeric ratio, resulting in even opposite selectivity.

**Scheme 54:**
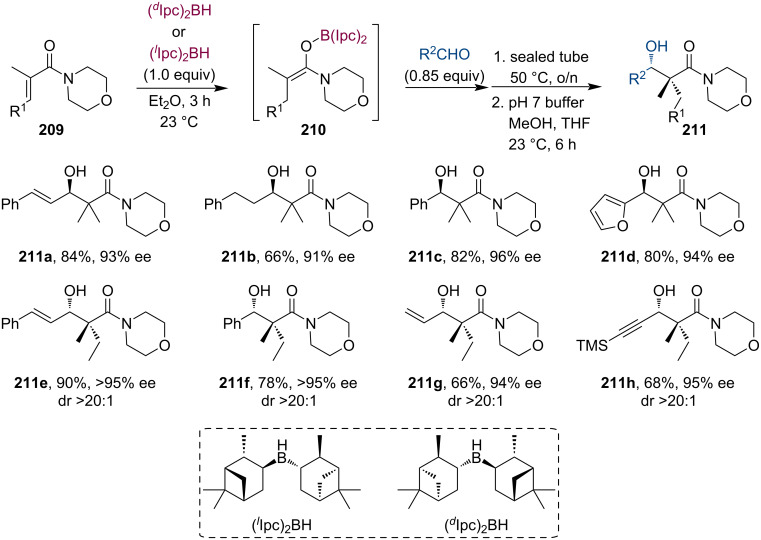
(Diisopinocampheyl)borane-promoted 1,4-hydroboration of α,β-unsaturated morpholine carboxamides and subsequent aldol reaction.

Roush and co-workers have presented a simple stereoselective reductive aldol procedure for the synthesis of tetrasubstituted enolates **210** from substituted morpholine acrylamides **209** (Scheme 54) [99]. Subsequent trapping of the boron enolate with various aldehydes provided the aldol adducts with good yields. Compared to general aldol reactions, the boron enolates showed lower reactivity and required overnight reflux to achieve good conversions. Nevertheless, the stereoselectivity of the reaction was still excellent (up to >95% ee, dr >20:1). Due to the high diastereoselectivity, the authors have concluded that the boron enolates are stable and do not isomerize by reversible formation of C–boryl species. The stereochemical information of the enolate is most likely transferred to the final product via a chair-like transition state.

### Application in total synthesis

**Figure 2:**
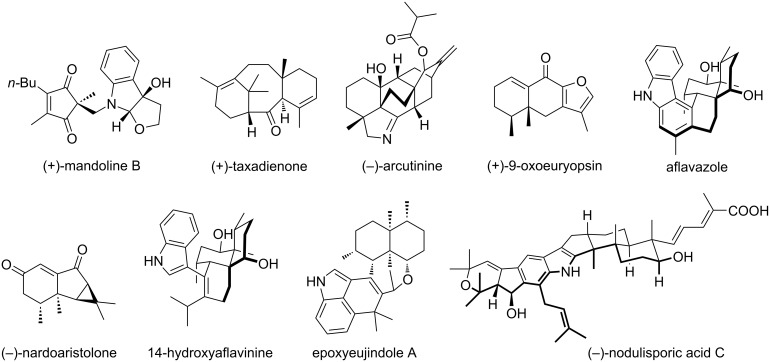
Some examples of total syntheses that have been recently reviewed.

As shown before, asymmetric tandem conjugate additions followed by enolate trapping are robust methodologies for synthesizing complex structures with multiple stereogenic centers. For this reason, such stereoselective procedures are commonly used in total synthesis (Figure 2) [100–101]. In this chapter, a few other examples are discussed.

**Scheme 55:**
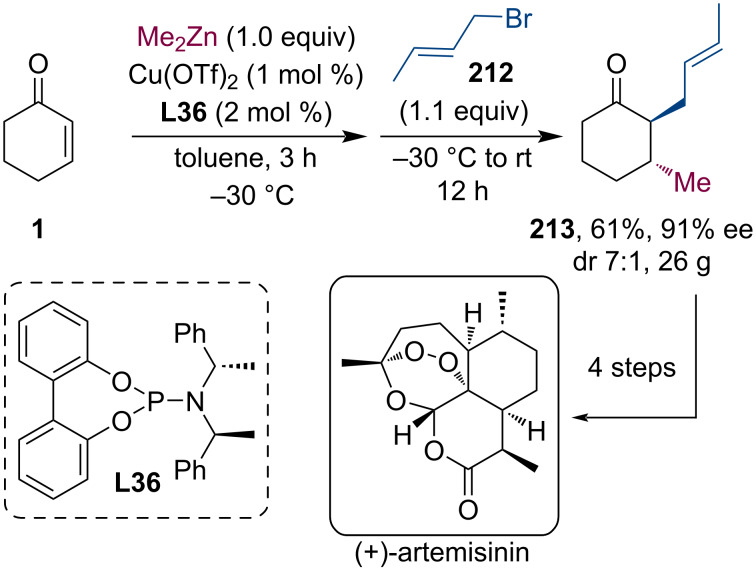
Stereoselective synthesis of antimalarial prodrug (+)-artemisinin utilizing a tandem conjugate addition/alkylation sequence.

Malaria is one of the most widespread diseases that still poses a severe threat to inhabitants and travelers of tropical regions within Africa, Asia, and Latin America. Uncomplicated cases, caused by *Plasmodium* parasites, are usually successfully treated by artemisinin combination therapy (ACT). Artemisinin can be isolated from the *Artemisia annua* (sweet wormwood) plant. This sesquiterpene lactone bearing a peroxide is a prodrug of the biologically active dihydroartemisinin. In 2012, Zhu and Cook developed a gram-scale asymmetric total synthesis of (+)-artemisinin (Scheme 55) [102]. Using the commercially available and cheap cyclohexenone **1** as starting material, they have demonstrated an economic synthesis plan in only five steps. In the first step, the Cu-catalyzed conjugate addition of Me_2_Zn is followed by alkylation with 1-bromobut-2-ene (**212**). The product of this tandem sequence was isolated on a multigram scale (26 g) in 61% yield and 91% ee with a *trans*/*cis* diastereomeric ratio of 7:1.

**Scheme 56:**
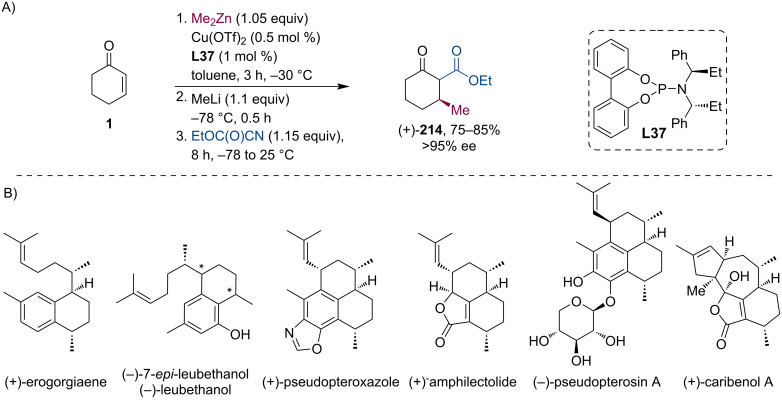
Amphilectane and serrulatane diterpenoids: preparation of chiral starting material via asymmetric tandem conjugate addition/acylation (A) and structures of prepared molecules (B).

Later, Luo and co-workers developed a modular, enantioselective synthetic approach to various amphilectane and serrulatane diterpenoids (Scheme 56B) [103]. These complex natural compounds exhibit strong pharmacological activities like anti-inflammatory, antituberculosis, analgesic properties, etc. The key reaction steps included a highly stereoselective gold-catalyzed or thermally activated Cope rearrangement and a gold-catalyzed 6-*endo*-*dig* cyclization. The chiral starting material was prepared by an asymmetric Cu-catalyzed tandem conjugate addition/acylation sequence using ethyl cyanoacetate (Mander’s reagent) as a trapping agent (Scheme 56A). Activation of the zinc enolate by MeLi was necessary, but with optimum reaction conditions the authors were able to isolate product (+)-**214** in good yield (75–85%) and excellent stereoselectivity (>95%) on a multigram scale. Additionally, both stereoisomers are available by simply using the ligand with the opposite stereochemistry.

**Scheme 57:**
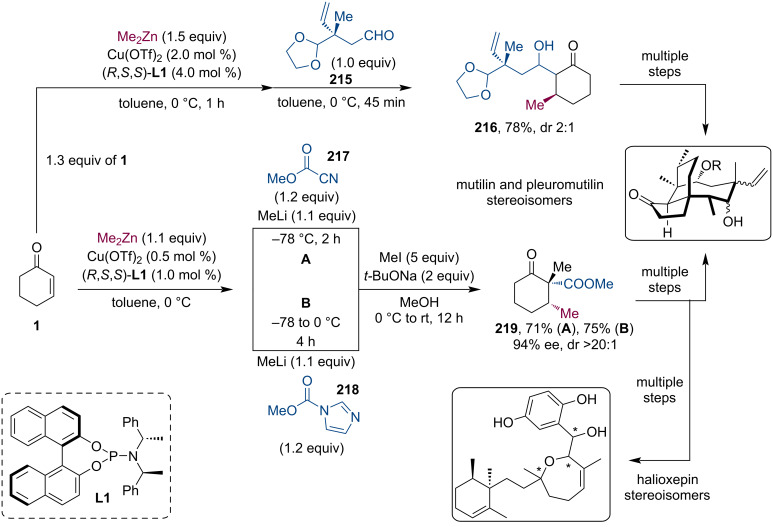
Various asymmetric syntheses of pleuromutilin and related compounds based on a tandem conjugate addition/acylation sequence. The same procedure provides access to halioxepin as well.

Pleuromutilin-based antibiotics are an essential line of defense in the war against resistant bacteria strains. The tricyclic diterpene fungal metabolite (+)-pleuromutilin was isolated in 1951 [104]. Since then it has served as a starting point for developing new antibiotics, including semisynthetic derivatives effective against Gram-positive or even both types of bacterial species. The C14 analogs, tiamulin, and valnemulin have been used by veterinarians since the 1980s. The topical antibiotic retapamulin was approved by FDA in 2007 for the treatment of the skin infection impetigo. In 2019–2020, lefamulin was introduced both in the USA and the EU to treat community-acquired bacterial pneumonia. Herzon et al. have demonstrated the modular synthesis of various pleuromutilins and created the foundation for the development of novel antibiotics against complicated infections. The key stereochemical information was usually introduced by a stereoselective tandem Cu-catalyzed conjugate addition and subsequent trapping of the zinc enolate by acylation or aldol reaction with an overall yield of 71–78% and good stereoselectivity (Scheme 57) [105–107]. Recently, Poock and Kalesse demonstrated the first total synthesis of halioxepine, a meridoterpene isolated from the Indonesian sponge *Haliclona* sp. [108]. Their synthesis takes advantage of the same tandem procedure that gives β-ketoester **219**. The asymmetric conjugate 1,4-addition and subsequent acylation provided good stereocontrol and the authors could revise the thus far incorrectly assigned configuration of the target compound.

**Scheme 58:**
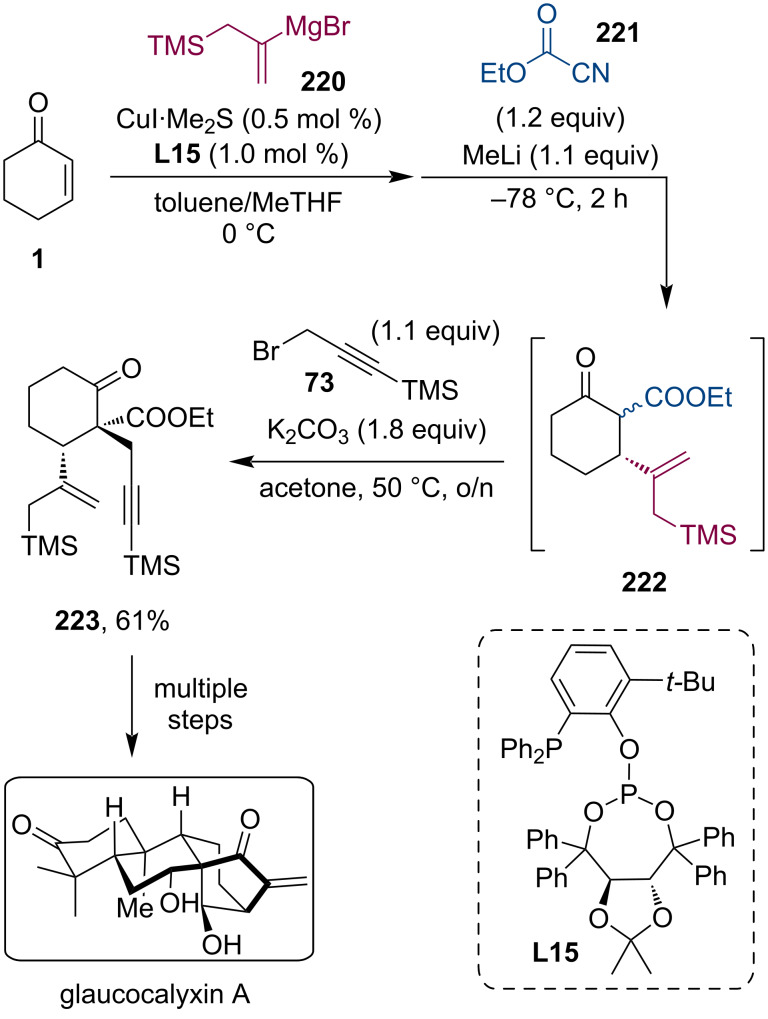
Total synthesis of glaucocalyxin A utilizing a tandem conjugate addition/acylation reaction sequence.

A similar tandem conjugate addition/acylation reaction sequence was utilized by the group of Jia in their work on the total synthesis of (−)-glaucocalyxin A [109]. Such diterpenoids, containing a 14-oxygenated bicyclo[3.2.1]octane ring system with several continuous stereocenters, are quite challenging targets for total synthesis, however, their biological properties render them highly valuable compounds. The authors utilized a Mn(OAc)_3_-mediated oxidative cyclization strategy, which begins with the introduction of the fundamental stereochemical information through an asymmetric tandem conjugate addition to cyclohexenone **1**, followed by the trapping of the Mg enolate with ethyl cyanoacetate (**221**). Consequent α-alkylation resulted in the multifunctionalized product **223** in 61% yield (Scheme 58).

**Scheme 59:**
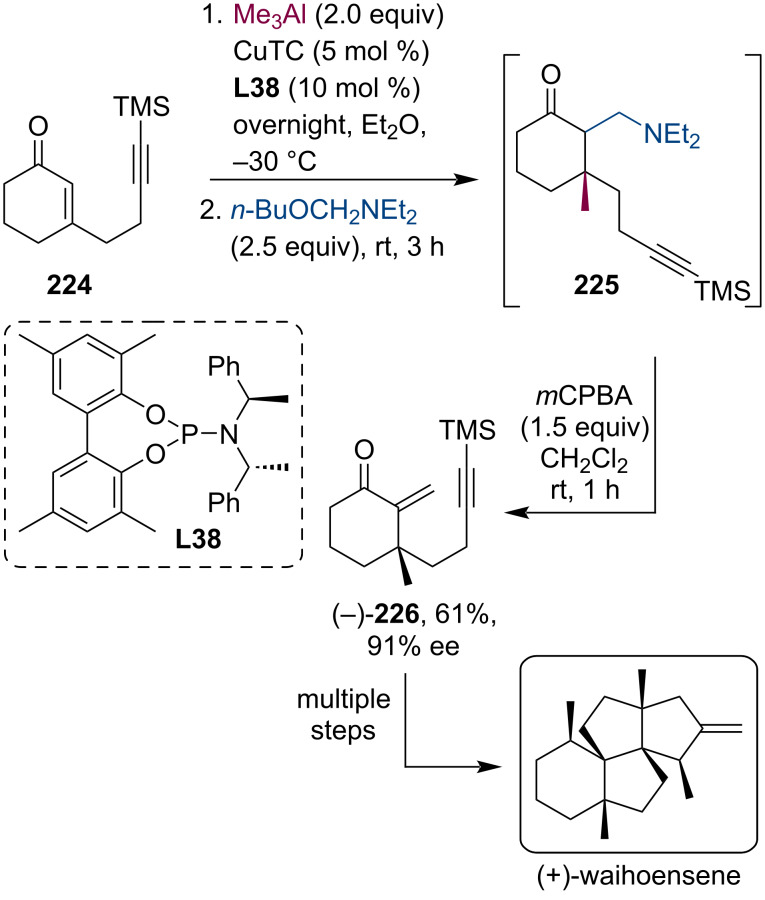
Installation of the exocyclic double bond using a tandem conjugate addition/aminomethylation sequence followed by oxidation during the total synthesis of waihoensene.

Natural products with complex multicyclic structures lacking functional groups (lack of oxygenation) are particularly difficult targets for synthetic chemists. Nevertheless, Huang and co-workers have successfully resolved the total synthesis of waihoensene which was isolated in 1997 from a New Zealand podocarp [110]. This diterpene has a unique substructure with fused 5-membered rings in an angular fashion and contains six contiguous stereogenic centers out of which four are all-carbon quaternary stereocenters. The authors have achieved the stereoselective synthesis of waihoensene for the first time with a 3.8% overall yield (15 steps) (Scheme 59). The construction of the triquinane core included a Cu-catalyzed asymmetric conjugate addition/aminomethylation followed by an oxidation to install the exocyclic double bond. The enone **226** was isolated in 61% yield and 91% ee.

**Scheme 60:**
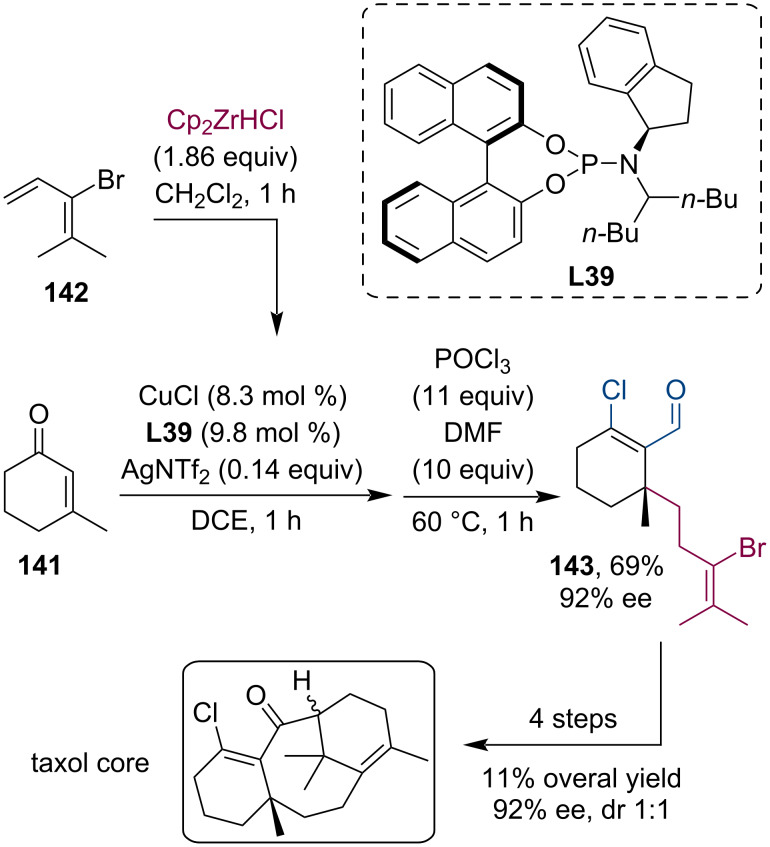
Synthesis of the taxol core using a tandem conjugate addition/enolate trapping sequence with Vilsmeier–Haack reagent.

Paclitaxel (taxol) is a highly successful chemotherapy medication that can be isolated from the Pacific jew tree (*Taxus brevifolia*), however, production from the natural source could hardly satisfy the high demand. Therefore, considerable effort was made toward the development of a cost-effective chemical production. In 2020, Fletcher and Wang joined the pursuit of a more efficient approach to produce taxol and related compounds [71]. Their methodology employed a multistep tandem procedure (Scheme 60): first, the alkylzirconium nucleophile was produced by hydrometalation of the functionalized alkene **142**. Next, this organozirconium reagent was used in a Cu-catalyzed asymmetric conjugate addition to 3-methyl-2-cyclohex-2-ene-1-one (**141**) followed by the trapping of the metal enolate with Vilsmeier–Haack reagent. This way, the β-chloroaldehyde **143** was isolated in 69% yield and 92% ee. Further transformation of this compound resulted in the taxol core in only 4 steps with 11% overall yield while retaining the correct stereochemistry introduced in the first step by the phosphoramidite ligand **L39** (92% ee, dr 1:1) (Scheme 60).

**Scheme 61:**
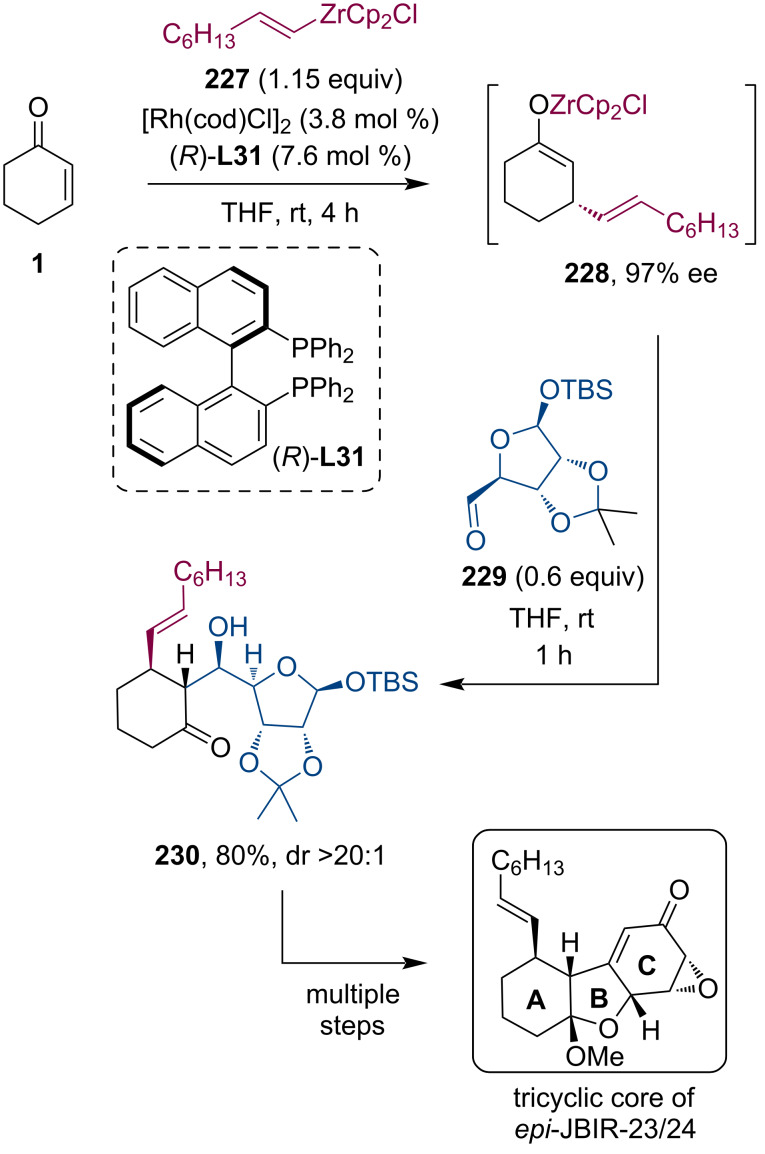
Synthesis of the tricyclic core of 12-*epi*-JBIR-23/24 utilizing a Rh-catalyzed asymmetric conjugate addition/aldol reaction tandem sequence.

Recently, Liu and co-workers reported the stereoselective synthesis of the tricyclic core of dodecahydrodibenzo[*b*,*d*]furan skeleton containing 12-*epi*-JBIR-23 and -24 [111]. Besides their intriguing complex structures, these novel compounds also found interest due to their potential inhibitory activity against malignant pleural mesothelioma (MPM) cell lines. Their synthetic route includes a key tandem sequence producing three neighboring stereocenters. Zirconium enolate **228** was prepared by the Rh-catalyzed conjugate addition of organozirconium reagent **227** to enone **1**. In the presence of the (*R*)-BINAP ligand, the Michael adduct **228** could be isolated in 97% ee. Finally, the Zr enolate was trapped by aldehyde **229** prepared from ᴅ-ribose. The aldol adduct **230** was isolated in 80% yield and excellent diastereoselectivity (>20:1). Additional transformation of compound **230** following an A–AB–ABC synthetic strategy resulted in the desired complex tricyclic skeleton opening the door for the total synthesis of 12-*epi*-JBIR-23/24 (Scheme 61).

**Scheme 62:**
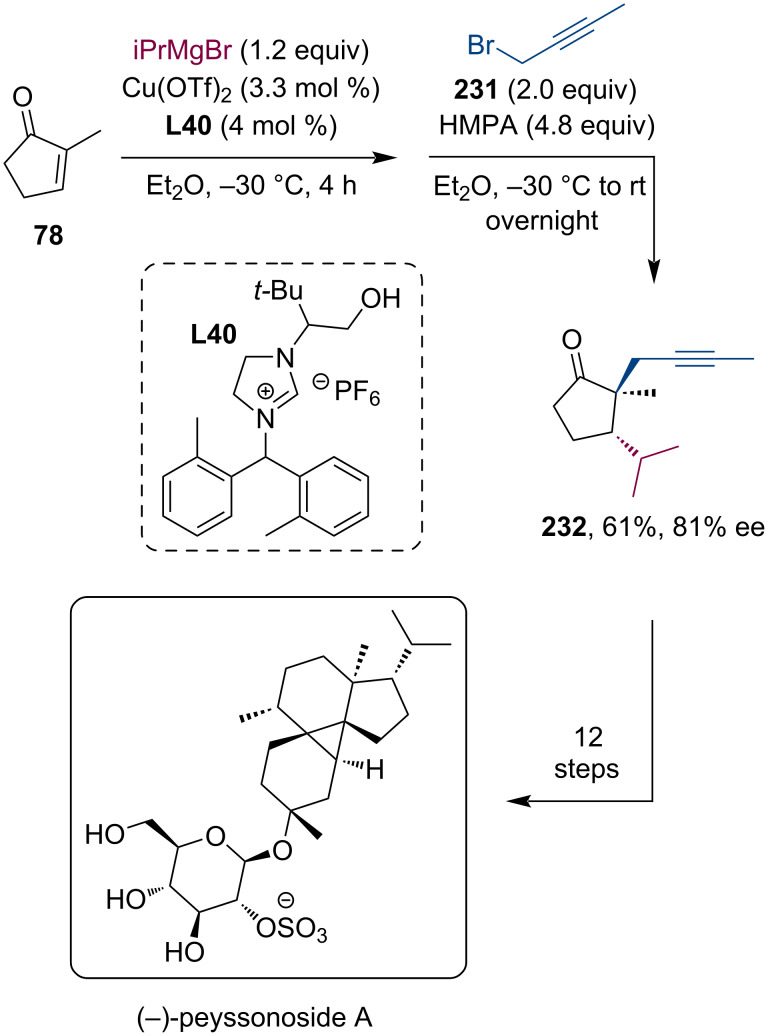
Total synthesis of (−)-peyssonoside A utilizing a Cu-catalyzed enantioselective tandem conjugate addition/enolate alkylation reaction sequence.

The sulfated β-glycoside peyssonnoside A was isolated only recently from the red algae *Peyssonnelia* sp. This diterpene showed promising biological activity against methicillin-resistant *Staphylococcus aureus* (MRSA) and liver-stage *Plasmodium berghei*. Structurally, peyssonnoside A belongs to a new class of diterpene glycosides with a distinctive tetracyclic carbon skeleton. From the point of view of a synthetic chemist, the most remarkable feature of this structure is the highly substituted cyclopropane ring incorporating two all-carbon quaternary centers, while the whole structure contains 7 stereocenters. Xu et al. recently demonstrated their 13-step total synthesis of (−)-peyssonnoside A, which begins with a Cu-catalyzed enantioselective conjugate addition/enolate alkylation tandem reaction sequence utilizing the N-heterocyclic carbene ligand **L40** [112]. Ketone **232** was isolated in 61% yield and 81% ee. Hereafter, the authors were able to synthesize the complex tetracyclic structure within only 12 additional steps, demonstrating how contemporary catalytic methodologies can facilitate the preparation of synthetically demanding natural products (Scheme 62).

## Conclusion

Tandem reactions based on asymmetric conjugate addition and subsequent enolate trapping reactions have undeniably matured into a robust methodology. The multitude of chiral ligands available for the Cu-catalyzed addition of organometallic reagents allows the efficient introduction of chirality and functionalization of various Michael acceptors as substrates. Typical polar organometallics such as dialkylzinc, Grignard, trialkylaluminum, or organozirconium reagents are suitable for fine-tuning the conjugate addition step and the generation of the corresponding metal enolates. Less reactive Michael acceptors can be advantageously activated by Lewis acids (e.g., TMSOTf and BF_3_·OEt_2_). These methods likely lead to silicon or boryl enolates that are also highly synthetically relevant nucleophiles. The methodology applies also to asymmetric conjugate silylation or borylation, which directly produces Si or B enolates. The high reactivity of metal enolates generated by these conjugate additions enables them to engage directly with various electrophiles such as carbonyl compounds, imines and their synthetic equivalents, Michael acceptors, alkyl halides, and carbenium ions. These trapping reactions allow rapid construction of molecules having high synthetic complexity and provide access to great structural variability in the final products. Unsurprisingly, tandem reactions of ACA-formed enolates were utilized in the syntheses of numerous natural products.

Newer advances in this methodology document that the synthetic community is continually pushing the limitations of these transformations, which paves the way to more exciting applications. Among the limitations of this methodology is the high basicity and reactivity of metal enolates formed in the conjugate addition step. This fact stems from the conjugate addition of polar and highly reactive organometallic species, which require careful handling and cryogenic conditions. The utilization of milder organozirconium reagents is an attempt to solve this issue. On the other hand, the high reactivity of metal enolates is advantageous for subsequent trapping reactions with electrophiles. We believe that here lies the possibility for further development. Finding more active catalysts would enable the conjugate addition of less reactive organometallic reagents. At the same time, it is necessary to identify suitable electrophilic reactions that would allow the use of less reactive enolates. The possibility for catalytic activation of these enolate trapping reactions still needs to be explored but it may hide undetected reactivities so far. Another problem is the stereoselectivity of the enolate trapping reactions. Chiral ligands import chirality on the initial Michael acceptor and the chirality of the enolate usually determines the stereoselectivity of the trapping reaction. However, stereoselectivities concerning enolate additions to carbonyl compounds or imines were often poor and new strategies are needed to address this problem.

Intriguing question is whether some less traditional activation techniques such as microwave, photocatalysis, flow chemistry or mechanical activation might not be applicable also to the reactivity of ACA-generated enolates. There are some hints that the use of polar organometallics might be possible and beneficial under these conditions.

In this review, we analyzed recent developments in the trapping reactions of chiral enolates obtained by conjugate additions. We have also highlighted our attempts to explore possibilities for enolate trapping reactions with unusual electrophiles. We hope that we helped researchers working in or interested in this area to navigate this fascinating field of research and stimulate further development of this methodology.
